# Mating of the Stichotrichous Ciliate *Oxytricha trifallax* Induces Production of a Class of 27 nt Small RNAs Derived from the Parental Macronucleus

**DOI:** 10.1371/journal.pone.0042371

**Published:** 2012-08-10

**Authors:** Alan M. Zahler, Zachary T. Neeb, Athena Lin, Sol Katzman

**Affiliations:** 1 Department of Molecular, Cell and Developmental Biology and The Center for Molecular Biology of RNA, University of California Santa Cruz, Santa Cruz, California, United States of America; 2 The Center for Biomolecular Science and Engineering, University of California Santa Cruz, Santa Cruz, California, United States of America; Sun Yat-sen University, China

## Abstract

Ciliated protozoans possess two types of nuclei; a transcriptionally silent micronucleus, which serves as the germ line nucleus, and a transcriptionally active macronucleus, which serves as the somatic nucleus. The macronucleus is derived from a new diploid micronucleus after mating, with epigenetic information contributed by the parental macronucleus serving to guide the formation of the new macronucleus. In the stichotrichous ciliate *Oxytricha trifallax*, the macronuclear DNA is highly processed to yield gene-sized nanochromosomes with telomeres at each end. Here we report that soon after mating of *Oxytricha trifallax*, abundant 27 nt small RNAs are produced that are not present prior to mating. We performed next generation sequencing of *Oxytricha* small RNAs from vegetative and mating cells. Using sequence comparisons between macronuclear and micronuclear versions of genes, we found that the 27 nt RNA class derives from the parental macronucleus, not the developing macronucleus. These small RNAs are produced equally from both strands of macronuclear nanochromosomes, but in a highly non-uniform distribution along the length of the nanochromosome, and with a particular depletion in the 30 nt telomere-proximal positions. This production of small RNAs from the parental macronucleus during macronuclear development stands in contrast to the mechanism of epigenetic control in the distantly related ciliate *Tetrahymena*. In that species, 28–29 nt scanRNAs are produced from the micronucleus and these micronuclear-derived RNAs serve as epigenetic controllers of macronuclear development. Unlike the *Tetrahymena* scanRNAs, the *Oxytricha* macronuclear-derived 27 mers are not modified by 2′O-methylation at their 3′ ends. We propose models for the role of these “27macRNAs” in macronuclear development.

## Introduction

Ciliated protozoans are characterized by nuclear dimorphism. These large single-celled organisms possess two types of nuclei; the macronucleus undergoes active transcription (the somatic nucleus) while the micronucleus, which is not transcribed, serves as the genetic repository (the germ line nucleus). During sexual reproduction, the parental micronuclei undergo meiosis and haploid micronuclei are exchanged between conjugating (mating) cells to form a new diploid micronucleus. This new diploid micronucleus divides by mitosis, and one of its daughter nuclei develops into the new macronucleus. This new developing macronucleus is referred to as the anlage. While the new macronucleus develops from the anlage, the parental macronucleus is destroyed. The process of macronuclear development from a diploid micronucleus requires extensive DNA amplification and polytenization, followed by elimination of micronucleus-specific sequences. This elimination results in fragmentation to smaller chromosomes, to which telomeres are added *de novo*. DNA elimination also occurs within macronuclear-destined regions resulting in splicing out of internally eliminated sequences (IESs) and rejoining of macronuclear-destined sequences (MDSs) (for general reviews of the process of macronuclear development see [1]–[4]).

Among the ciliates, the stichotrichous ciliates (including *Stylonichia*, *Euplotes* and *Oxytricha* species), take the processing of micronuclear sequences into macronuclear chromosomes to an extreme. Using re-association kinetics, it was measured that the macronuclear genome possesses only 5% of the sequence complexity of the micronuclear genome [5]. While the micronuclear chromosomes of this group are typical of eukaryotes in terms of chromosome length, structure and mitotic division, the macronuclear genome consists of over 20,000 different chromosomes with an average length of ∼2,200 bp [6]. The majority of these “nanochromosomes” contain only a single gene [7], [8] and are present in over 1000 copies per nucleus [5]. The micronuclear versions of macronuclear *Oxytricha* genes are not capable of being expressed without extensive DNA processing that occurs during macronuclear development. This includes the removal of internally eliminated sequences (IESs) followed by splicing together of the surrounding macronuclear destined sequences (MDSs). These IESs total over 100,000 in number [7]. At the junctions at which the MDSs are joined, there are short direct repeats referred to as “pointers”. Only one copy of each pointer pair from the micronuclear DNA is found in the macronucleus, suggesting a potential role for a homology-directed DNA repair mechanism in the process of IES elimination [9]. In addition to IES elimination, an added complexity of macronuclear development in the stichotrichous ciliates is that some genes have a different linear ordering of MDS segments in the micronucleus than in the macronucleus. The actin gene was the first of these scrambled micronuclear genes to be identified, and it is interesting in that MDS segments are not only out of order in the micronuclear genome, but MDS2 is actually found on the opposite strand from the others [10]. The alpha telomere binding protein and DNA polymerase alpha are two other highly scrambled micronuclear genes that have been characterized [11], [12].

In stichotrichous ciliates, and in the Oligohymenophorea ciliates, which include *Tetrahymena* and *Paramecium*, there is strong evidence that the parental macronucleus provides information to guide the developing macronucleus [4], [13]. In essence, the parental macronucleus serves as an epigenetic guide to daughter macronuclear formation. In both groups of ciliates, RNA serves as the mediator of epigenetic control [14]. In *Tetrahymena*, the diploid micronucleus is transcribed bi-directionally [15], and transcripts are processed by a dicer homolog, Dcl1p, to produce a 28–29 nt long class of small RNAs, called scanRNAs [16]–[18]. These scanRNAs are loaded into a complex with a Piwi homolog called Twi1p [17], [19]. The scanRNA complexes are sent to the parental macronucleus, where those that have a match to sequences in the parental macronucleus are selectively removed from the pool that will guide IES excision [4], [13]. Those that survive this parental macronuclear filtering step are then imported to the developing macronucleus, where they direct chromatin modifications that target micronuclear-specific sequences for degradation [4], [13], [20]–[22]. In the stichotrichous ciliate *Oxytricha trifallax*, it has been demonstrated experimentally that long RNAs are produced bi-directionally from the nanochromosomes of the parental macronucleus soon after mating. Experimental injection of single-stranded RNAs into the cytoplasm of mating cells can reprogram IES removal and MDS joining, suggesting that these long RNAs serve as a guide for MDS joining and gene unscrambling [23]. The resulting nanochromosomes are differentially amplified in the developing macronucleus. Epigenetic information concerning nanochromosome amplification levels in the parental macronucleus is transferred to the developing macronucleus via RNA intermediates [24], [25].

We tested for the presence of small RNA species in the stichotrichous ciliate *Oxytricha trifallax* that are specifically produced during mating. We identified a class of 27 nt long RNAs that are expressed at high levels 24 hours after mating induction, and which decrease steadily during the subsequent steps of macronuclear development. While the scanRNAs of *Tetrahymena* are modified with a 2′O-methyl group at their 3′ ends [26], the 27 nt class produced in *Oxytricha* have 2′ and 3′ hydroxyl groups at their 3′ ends. We performed next generation sequencing of small RNAs from vegetative cells and mating cells over a time course after mixing cells of mating-competent strains. We demonstrate that the 27 nt RNAs originate from macronuclear chromosomes and are transcribed from both strands. Their distribution along the nanochromosomes is non-uniform, and for either strand the positions proximal to the telomeric repeats are much lower in small RNA coverage. We name these 27 nt mating-specific small RNAs “27macRNAs”. We propose several models for the roles that 27macRNAs may play during macronuclear development.

## Results

### *Oxytricha trifallax* produces a class of 27 nt small RNAs during macronuclear development

**Figure 1:**
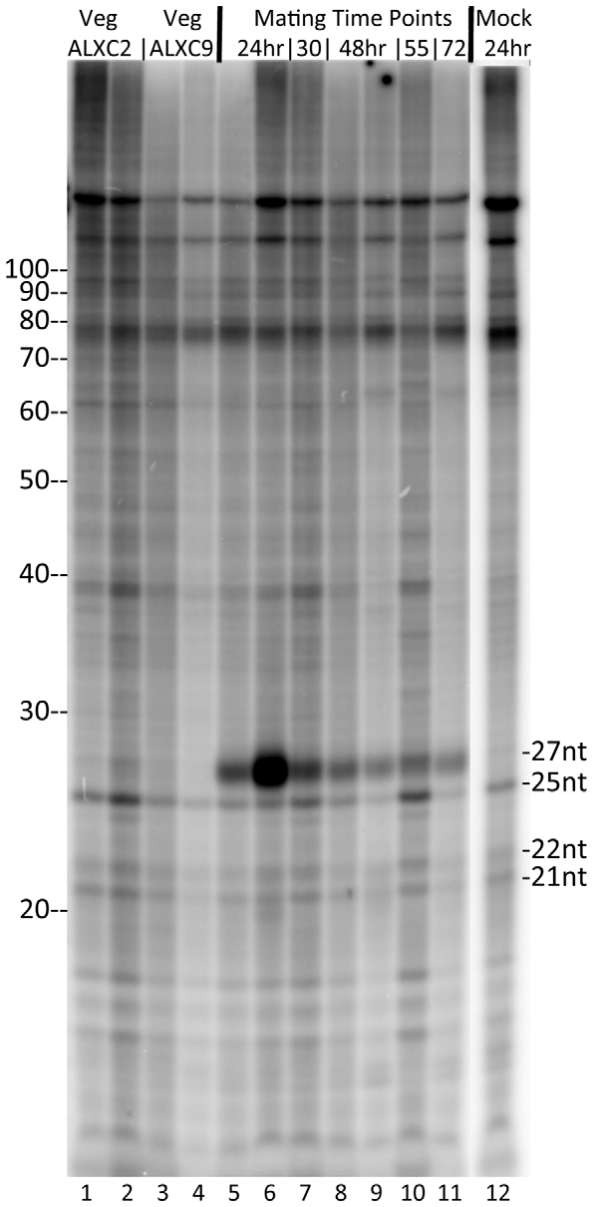
Mating in *Oxytricha trifallax* leads to production of a class of 27 nt RNAs. Total RNA was purified from vegetative *Oxytricha trifallax* (lanes 1–4) or at various time points after mixing together complementary mating strains (lanes 5–11). In addition, total RNA was purified from strain ALXC9 treated under identical conditions as a mating for 24 hrs (lane 12 - Mock 24hr). Total RNA was phosphatase treated followed by 5′ end labeling with ^32^P, separated on a 15% polyacrylamide denaturing gel and visualized using a PhosphorImager. Sizes from Decade RNA 10 nt Ladder (Ambion) are indicated at left. Positions of small RNAs of interest are indicated at right. Lanes 1–11 directly correspond to RNA preparations used to prepare libraries for Illumina sequencing listed in the same order in Table 1.

Small RNAs known as scanRNAs play an important role in the DNA rearrangements that occur during the development of the macronucleus in *Tetrahymena* [4], [13]. We asked whether small RNA species are present in vegetative stichotrichous ciliates and in those undergoing macronuclear development. To do this, total RNA was isolated from *Oxytricha trifallax* vegetative cells or cultures of cells in which complementary mating types had been mixed together under mating conditions. Total RNA was treated with calf intestine alkaline phosphatase and then ^32^P 5′ end labeled using gamma-^32^P-ATP and T4 polynucleotide kinase. Labeled RNAs were separated on a 15% acrylamide sequencing gel and visualized with a PhosphorImager. As seen in Figure 1, a prominent band at 27 nt appears 24 hours after mixing of the mating competent strains. This band is not detectable 5, 6 or 12 hours after mixing of the mating competent strains (data not shown), but by 24 hours it is quite prominent. At later stages of macronuclear development, the relative intensity of this band fades. Three other bands are faint but detectable in all RNA isolations. These RNAs are 25, 22 and 21 nt in size. As these are not induced by mating, these are not a major focus of this paper. We did a control experiment to confirm that the labeled RNAs were from *Oxytricha* (and not a contaminant from the algal food source) by labeling an equivalent amount of total RNA from the *Chlorogonium elongatum* food source (data not shown). Equivalent amounts of total Chlorogonium RNA, labeled and run in parallel lanes, did not yield detectable bands at these sizes. We also did a mock mating with the ALXC9 strain, starving it and moving it into Pringsheim buffer for 24 hours, to test whether these 27 nt RNAs are induced by starvation as opposed to mating. This treatment, equivalent to the mating procedure but done with only one strain that does not self mate, did not lead to production of 27 nt small RNAs (Figure 1, lane 12).

### Mating-specific small RNAs have 2′ and 3′ OH groups at their 3′ ends

**Figure 2:**
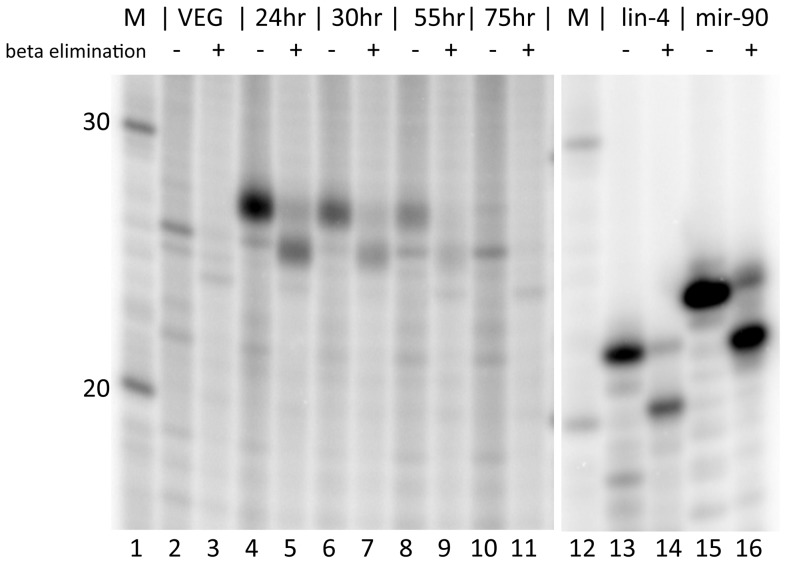
The mating-specific 27 nt RNAs in *Oxytricha trifallax* are not modified at their 3′ ends. RNAs were tested with the beta elimination assay in order to determine if there is a modification at the 3′ end. RNAs tested are ^32^P 5′ end labeled total *Oxytricha trifallax* RNA (lanes 2–11) or 5′ end labeled synthetic positive control RNAs containing the sequence of the *C. elegans lin-4* (21 nt) or *mir-90* (23 nt) microRNAs which were subsequently mixed with 1.5 µg of unlabeled *Oxytricha* total RNA prior to beta elimination (lanes 13–16). Control untreated samples (-) or samples subjected to the beta elimination reaction (+) are indicated. RNAs were isolated from vegetative ALXC2 (lanes 2 and 3) or from a mating between ALXC2 and ALXC9 that were harvested at the indicated timepoint after mixing (lanes 4–11). Beta elimination will remove the terminal ribose if both a free 2′ OH and 3′ OH are present, and will leave a 3′ cyclic phosphate. This loss of a nucleotide and the presence of the extra phosphate group will result in the treated RNAs running almost two bases faster than their untreated counterparts.

Having demonstrated that *Oxytricha* produce a 27 nt species of small RNAs during mating, we asked whether these are similar to the 28–29 nt long scanRNAs that are produced during mating by the distantly related ciliate *Tetrahymena*. One distinguishing feature of scanRNAs is that they have a 2′O-methyl group added to the 3′ terminal ribose of the RNA by a *Tetrahymena* homolog of the Hen1p methylase, and this activity is essential for scanRNA stability and function [26]. We performed a beta elimination assay to determine if there are modifications present at the 3′ end of the 27 nt *Oxytricha* mating-specific RNA species. If free 2′ OH and 3′ OH groups are present at the 3′ terminal ribose of RNA, then the ribose can be oxidized by periodate and subsequently removed during incubation at higher pH and temperature, resulting in an increase in mobility on a sequencing gel [27]. Figure 2 shows the results of this assay. The 27 nt mating-specific RNA species is clearly modified by the beta elimination assay (lanes 4 through 8), in a way that is similar to the control RNAs (lanes 13–16). The beta elimination reaction results in the removal of the 3′ nucleotide, and leads to RNAs ending in a 3′ cyclic phosphate; the increased charge per length ratio contributed by the 3′ cyclic phosphate leads to these modified RNAs appearing to run 2 bases faster than their untreated controls. This result suggests that the 27 nt RNA has different properties than the 28–29 nt scanRNAs induced by mating in *Tetrahymena*. The 25 nt RNA species is sensitive to beta elimination as well (lanes 2 and 3, and 10 and 11), indicating that it too lacks modifications of the terminal ribose.

### Small RNA Sequencing and Analysis

**Figure 3:**
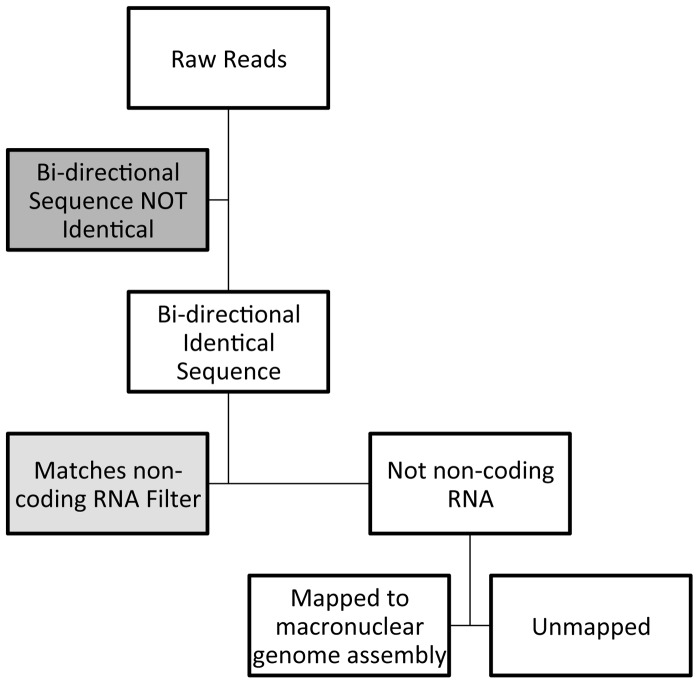
Flowchart of sequence analysis.

In order to better understand the small RNA species observed in Figure 1, we performed high-throughput sequencing of the small RNA in these samples. Libraries of small RNAs were prepared for sequencing using the TruSeq small RNA Sample Prep Kit (Illumina) starting with 1–2 micrograms of total RNA from preparations shown in lanes 1–11 of Figure 1. The cDNA library was prepared by the manufacturer's standard protocol, which required that small RNAs possess a 3′OH group and a 5′ monophosphate in order to be ligated to the adapters. After linker attachment and amplification, equal amounts (2 ng) of cDNA representing small RNAs of 15–45 nt in length (as determined by BioAnalyzer (Agilent) analysis) were pooled, and the proper sized cDNAs selected and sequenced in one lane of an Illumina HiSeq2000 Sequencer at the UCSC Genome Technology Center using a 100 base paired-end read protocol. Figure 3 shows a flowchart of the sequence analysis for each library and Table 1 shows our initial analysis of the sequences in each library. Taking advantage of the fact that our lane was on a slide that underwent bi-directional sequencing, and that the lengths of the RNAs under investigation (<45 nt) were shorter than the sequencing length (100 bp), we only processed sequences that were identical in both directions. We used the data from a careful analysis of non-coding RNAs encoded in the *Oxytricha trifallax* macronucleus [28] to assemble a filter for non-coding RNAs. The percentage of bi-directional identical reads in each library that derived from non-coding RNA fragments varied between 13% and 43%. Ciliates possess two genomes, a micronuclear genome and a macronuclear genome. While very limited micronuclear sequence is available for *Oxytricha trifallax*, an extensive but incomplete assembly of macronuclear sequence data called WGS2.1.1 is available [28]. From those sequence data, we extracted the sequences of 10,137 full-length telomere-to-telomere nanochromosome sequences. Given the short nature of nanochromosomes, we decided to concatenate these sequences together into one longer file with 50 Ns inserted between each full-length nanochromosome. An additional 46,417 contigs of incomplete nanochromosome sequence were extracted from WGS2.1.1 and these were also concatenated with 50 Ns inserted between each contig. We also compared the small RNA reads to the published 70 kb mitochondrial genome sequence. The percentage of sequence reads that survive the non-coding RNA filter and map to these sequences are also indicated in Table 1. An initial analysis of the mappings to the mitochondrial genome identified a denser mapping of short RNA reads to the mitochondrial genome relative to the macronuclear genome. However, our initial cursory analysis found that the majority of these mappings were fragments of mitochondrial tRNAs; many classes of mitochondrial non-coding RNAs were not filtered by the non-coding RNA filter step (those non-coding RNAs were encoded by the macronuclear genome). Given the poor mitochondrial non-coding RNA filtering, and the finding that the abundant 27 nt reads from RNAs derived from mating cells were insubstantial in their mapping to the mitochondrial genome (976 of the distinct 27 nt reads matched mitochondrial genome vs. 2.7 million distinct 27 nt reads matching the macronuclear sequence assembly in a 24 hour mating library), we decided not to pursue an analysis of the remaining small RNAs that matched the mitochondrial genome.

**Table 1:** *Oxytricha trifallax* small RNA sequencing mapping statistics.

Library Name	Raw Reads	Bi- directional Identical Reads	Matches to ncRNA Filter	ncRNA%	Not ncRNA	Not ncRNA %	No macro-nuclear match	Mapped to Macro- nucleus	Mapped to Macro- nucleus%	> = 10 Map Sites	<10 Multi Map Sites	Unique Map Sites
veg02_01	12.64M	9.68M	3.62M	37.50%	6.05M	62.50%	2.68M	3.38M	55.80%	0.17M	1.55M	1.66M
veg02_02	11.02M	8.37M	1.59M	18.90%	6.79M	81.10%	3.59M	3.19M	47.10%	0.05M	1.40M	1.75M
veg09_03	8.71M	6.42M	0.84M	13.00%	5.58M	87.00%	4.30M	1.28M	23.00%	0.02M	0.58M	0.68M
veg09_04	8.28M	6.28M	1.64M	26.10%	4.64M	73.90%	3.05M	1.59M	34.20%	0.04M	0.73M	0.83M
mat24_05	9.16M	7.34M	1.13M	15.40%	6.21M	84.60%	2.25M	3.96M	63.70%	0.13M	1.42M	2.41M
mat24_06	13.20M	10.57M	1.55M	14.60%	9.02M	85.40%	3.11M	5.91M	65.50%	0.16M	2.11M	3.64M
mat30_07	12.39M	9.66M	2.90M	30.10%	6.75M	69.90%	2.53M	4.22M	62.50%	0.37M	1.46M	2.39M
mat48_08	9.59M	7.56M	1.90M	25.20%	5.66M	74.80%	2.31M	3.35M	59.10%	0.16M	1.18M	2.00M
mat48_09	7.32M	5.67M	1.58M	27.90%	4.09M	72.10%	1.77M	2.32M	56.60%	0.11M	0.88M	1.33M
mat55_10	10.74M	8.26M	3.59M	43.40%	4.67M	56.60%	1.86M	2.80M	60.10%	0.28M	0.95M	1.57M
mat72_11	8.43M	6.63M	2.49M	37.60%	4.14M	62.40%	1.79M	2.34M	56.80%	0.15M	0.82M	1.37M

Small RNA libraries were prepared from 11 different biological samples indicated under “Library name”. veg02 is vegetative ALXC2 and veg09 is from vegetative ALXC9. mat## libraries were made from matings between ALXC2 with ALXC9, and the time in hours after mixing of the strains that the RNA for the library was isolated is indicated (mat24 RNA was extracted 24 hours after mixing mating strains). “Raw Reads” are the number of bidirectionally sequenced reads for each library (M = million). “Bidirectional Identical Reads” are the number of reads that were identical in both directions. These were used as high confidence sequences for further mapping. The bidirectional identical reads were filtered through a collection of *Oxytricha trifallax* non-coding RNAs (ncRNAs). “Matches to ncRNA Filter” are the number of sequences that match ncRNAs, and ncRNA% are the percentage of bidirectional identical reads that match the ncRNA filter. “Not ncRNA” are reads that did not match the ncRNA filter, and “Not ncRNA%” are the percentage of Bidirectional Identical Reads that are not a match to ncRNA. Bowtie was used to align the Not ncRNA reads to the macronuclear genome chr1 and chr2, as well as the 70 kb mitochondrial genome (chrM). “No macronuclear match” is the number of reads that did not match to the macronuclear sequences. “Mapped to Macronucleus” indicates the number of reads that mapped to the macronuclear sequences, and “Mapped to Macronucleus %” shows the fraction of “not ncRNA” reads that are macronuclear in origin. “> = 10 Map Sites” are the number of reads that map to 10 or more places in the macronuclear sequence assembly, “<10 Multi Map Sites” are the number of reads that map to between 2 and 9 places in the macronuclear sequence assembly, and “Unique Map Sites” are the number of reads that map to only one position in the macronuclear sequence assembly.

### Analysis of size classes of small RNAs

**Figure 4:**
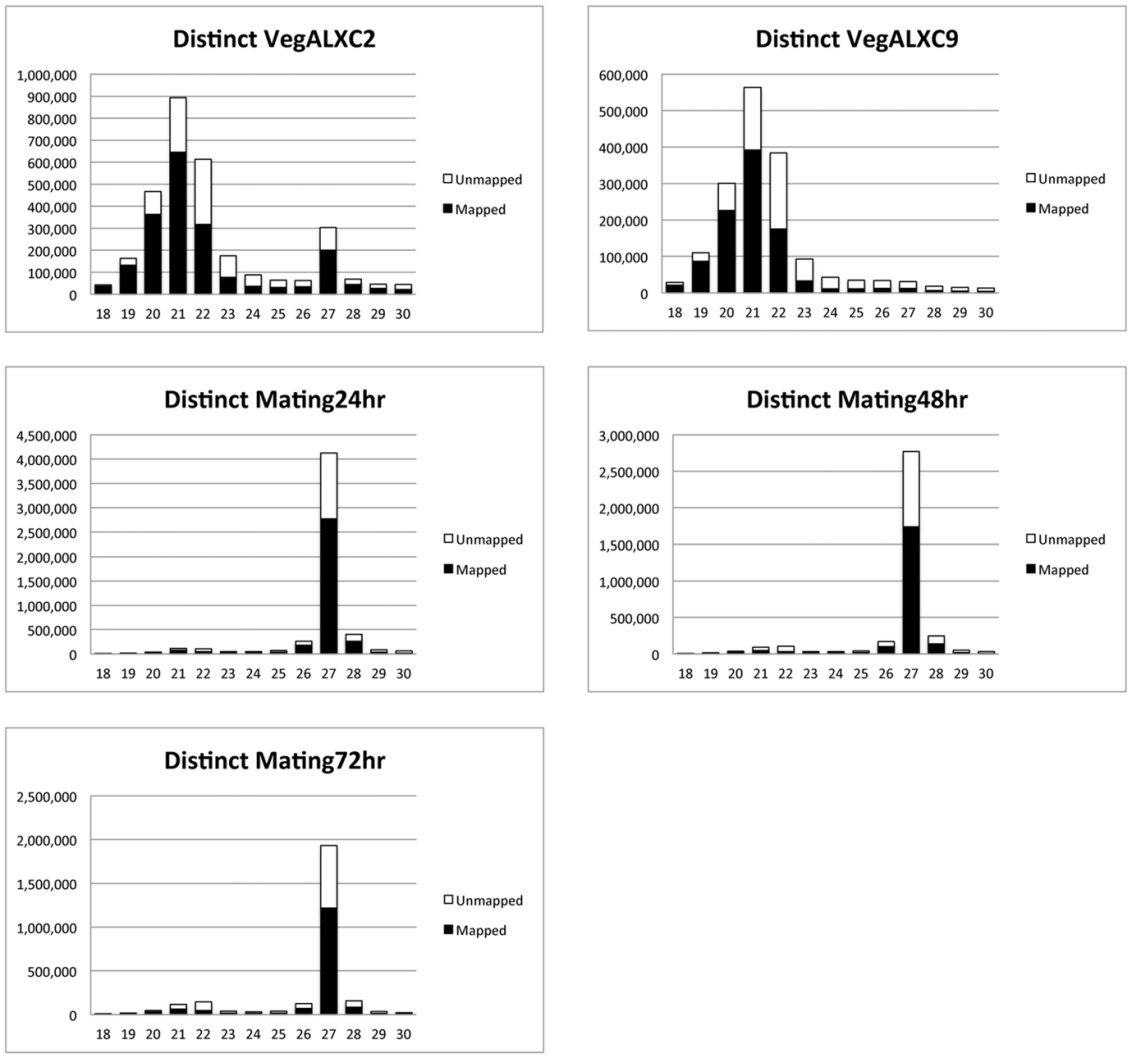
Vegetative small RNA libraries contain mostly 20–22 nt RNAs and mating libraries contain mostly 27 nt RNAs. The length distributions of sequencing reads in the small RNA range (18–30 nt) are plotted for five of the 11 libraries (comprising one replicate from each cell type or timepoint). The histograms are for distinct reads; only one occurrence of a sequence that appears more than once in a library is counted. The five representative libraries shown can be cross-referenced to table 1; VegALXC2 - veg02_01, VegALXC9 - veg09_04, Mat24hr - mat24_06, Mat48hr - mat48_08, Mat72hr - mat72_11. Black bars represent reads that mapped to the macronuclear sequence assembly. White portions of the bars represent reads that did not map to the macronuclear assembly. The vegetative strain VegALXC2 is capable of self-mating, leading to a minor peak at 27 nt.

For many small RNAs studied to date, different functional classes are characterized by their length. In order to look at this further, we created histograms of size distributions for RNAs that survived the non-coding RNA filter step (Figure 4). We generated length histograms from distinct sequence reads in each library (only one read was plotted if that exact sequence and length occurred for multiple reads in the library). We further analyzed the size distribution for the ability of reads to be mapped to the macronuclear sequence assembly. Several conclusions can be drawn from viewing these histograms. The 20 nt, 21 nt and 22 nt species predominate in vegetative cells while the 27 nt species is the predominant size in RNAs sequenced from mated cells. One interesting observation from looking at these histograms is the lack of any peak in any of the libraries for a 25 nt RNA species, which our ^32^P-labeling results indicate are present in all samples, both vegetative and mating (Figure 1). The cDNAs in the library were prepared by a protocol that required a 3′OH group and a 5′PO_4_ on the small RNAs for adapter ligation. The 25 nt species is not modified at its 3′ end (see lanes 2 vs. 3 and 10 vs. 11 in Figure 2). This RNA cannot be labeled by T4 polynucleotide kinase without first treating with calf intestine alkaline phosphatase (data not shown) suggesting that it has phosphate groups at its 5′ end. We suggest that the inability to recover these 25 nt RNAs in the library may be due to the presence of multiple phosphates at the 5′ end, which has been observed for *C. elegans* secondary silencing RNAs [29], [30].

Analysis of the histograms indicates distinct properties of the 20 nt and 21 nt classes vs. the 22 nt class of small RNA that predominate in the vegetative libraries. For distinct reads in the four vegetative libraries sequenced, 75.5±2.5% of the 20 mers and 70.0±2.2% of the 21 nt reads map to the macronuclear sequence assembly. Given that the macronuclear sequence assembly is incomplete, this indicates a strong likelihood that the 20 mers and 21 mers are macronuclear in origin. In contrast, only 46.6±5.1% of the distinct 22 nt reads from the vegetative libraries map to the macronuclear sequence assembly. This suggests the possibility that a substantial number of the 22 nt RNAs may arise from micronuclear sequences.

**Figure 5:**
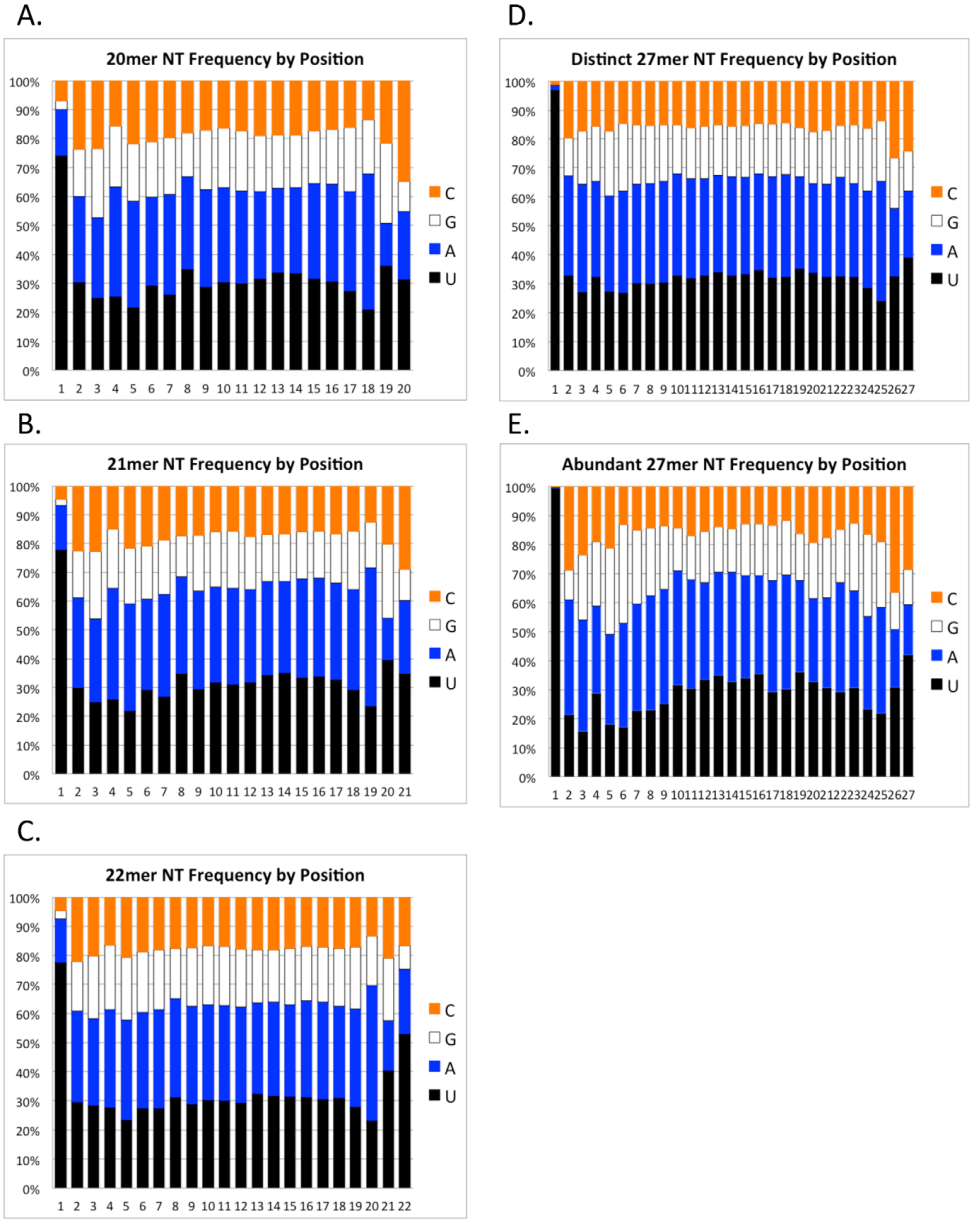
Nucleotide position bias in the different classes of sequenced small RNAs. These charts show the nucleotide frequency at each position for different small RNA size classes. The distinct sequences in the veg09_03 library and the mat24_05 library were filtered against non-coding RNAs. The distinct RNAs that made it through the filter were selected by size and the nucleotide composition of each position for the indicated size class was determined. **A**. Distinct 20 mers from the veg09_03 library (222,272 sequences). **B**. Distinct 21 mers from the veg09_03 library (416,2227 sequences). **C**. Distinct 22 mers from the veg09_03 library (327,422 sequences). **D**. Distinct 27 mers from the mat24_05 library (2,919,225 sequences). **E**. Abundant 27 mers from mat24_05 library comprising distinct sequences that were detected 10 or more times in the library (17,981 sequences).

We performed an analysis to look at the 5′ nucleotide identity of these classes of small RNAs. Several classes of small RNAs in *C. elegans*, such as 21U and 26G, are distinguished by a distinct 5′ nucleotide and length [31]. In an analysis of the 20, 21 and 22 nt RNAs from one of the vegetative libraries (Figures 5A, 5B and 5C), and the 27 nt RNAs from a 24 hour mating library (Figure 5D), we found that there is a strong bias towards U as the nucleotide at the 5′ end of each of these RNA classes. For the 20 nt species, 74.2% of the reads start with U, for the 21 nt species, 78.0% of the reads start with U, for the 22 nt species, 77.7% of the reads start with U and for the 27 nt species, 96.9% of the reads start with U. No other striking biases are observed at other positions, with the exception that over 50% of the 22 mers end in U. This preponderance of a 3′ terminal U is unique among the size classes. For the 27 mers, we asked whether the more abundant members of the group, comprising 17981 distinct sequences found between 10 and 100 times in the mat24_05 library, had a different nucleotide frequency at certain positions relative to the total pool of 2,919,225 distinct 27 mers in the same library (Figures 5D vs. 5E). With the exception of an increase in the frequency of U in the first nucleotide position from 96.9% in the total 27 mer pool to 99.4% in the abundant 27 mers, and a slight decrease in U frequency for nucleotides 2 through 9, there were no obvious sequence bias differences between these sets.

The 27 nt RNA species, whose production was induced by mating (Figure 1) is, as expected, the most prominent size class in all of the libraries made from mated cells. This 27 nt species can also be detected at a low level in ALXC2 vegetative cells but not in ALXC9. The ALXC2 strain undergoes a low level of self-mating under vegetative growth conditions while the ALXC9 strain does not. Therefore we conclude that production of the 27 nt RNA species is induced by mating. We find that between 63% and 67% of the distinct 27 nt reads in the mating libraries map to the macronuclear genome assembly. Given that this is an incomplete macronuclear genome assembly, and given that the macronucleus contains only 5% of the sequence complexity of the micronucleus, this implies that the majority of 27 nt RNAs are derived from macronuclear sequence. Given that the production of these RNAs peaks 24 hours after mixing of mating competent cells, which would correspond to an early polytene chromosome stage of macronuclear development in stichotrichs [31], [32], two hypotheses could explain their origin. These RNAs could originate from the parental macronucleus before it becomes degraded. Alternatively, these RNAs could be derived from the developing macronuclear anlage, having been transcribed specifically from the subset of DNA sequences that are destined to be retained in the new macronucleus.

### 27 nt RNAs originate from the parental macronucleus

In order to distinguish whether the 27 nt mating-specific RNAs originate from the parental macronucleus or the developing macronucleus, we aligned the 26–28 nt RNAs that survived the non-coding RNA filter against the micronuclear and macronuclear sequences for 6 different genes. We were limited in this analysis to six genes, because these are the only genes with complete micronuclear and macronuclear sequence pairs of the same gene alleles (there is very little micronuclear sequence available in public databases). We used the micronuclear and macronuclear sequences as targets for mapping and counted the number of sequence hits to each gene of either nuclear origin. Micronuclear and macronuclear gene sequences compared in this study were trimmed so that sequence length differences for each pair are only due to the presence of IESs and pointer sequence duplication in the micronuclear version. Table 2 shows the alignment statistics for all distinct 26–28 nt RNAs from the seven mating libraries. We treated this as a Venn diagram, determining the number of distinct 26–28 nt reads in the libraries that align to both the micronuclear and macronuclear versions, and the number that align to only one of the two versions. Alignment of a sequence only to the micronuclear version of a gene could be the result of alignment to an IES or an IES/MDS junction, which are unique to the micronucleus. Alignment to only the macronuclear sequence could be a result of an alignment that spans an MDS junction by extending past both sides of the “pointer” sequence. For all six genes tested, the majority of 26–28 nt reads align with both sequences. The number of reads that align with only the macronuclear version far exceeds the number of reads that align to only the micronuclear version for all six genes (see last four columns of Table 2). For example, the micronuclear version of the alpha telomere binding protein gene (TEBPAlpha) has 17 scrambled MDSs [11]. 996 reads aligned to both the micronuclear and macronuclear versions of this gene. 252 reads aligned only to the macronuclear version of the gene while only 4 reads aligned only to the micronuclear version of the gene. Since for each MDS/MDS junction, the micronuclear version of the gene contains more unique sites for alignment than the macronuclear version (the micronuclear version has the entire IES plus the two MDS/IES junctions as unique sequence while the macronuclear version only has the MDS/MDS junction as unique sequence), the bias towards macronuclear-specific reads is even more striking. The macronuclear genome only contains 5% of the complexity of the micronuclear genome but its nanochromosomes are amplified relative to the micronuclear genome. This could account for the relative paucity of micronuclear-specific reads even if the micronuclear genome is also transcribed into 27 mers. We controlled for that possibility by only mapping distinct sequences, as opposed to total sequence reads, to avoid multiple counting of sequences that derive from amplified nanochromosomes in the macronucleus. Taken together, the data in Table 2 indicate that the majority of the 26–28 nt RNA species in mating cells originate from the parental macronucleus. The small number of micronuclear-specific reads for scrambled genes in Table 2 suggest that there may be a potential for some micronuclear production of 27 nt RNAs. Alternatively, since all of the micronuclear-matching sequences came from the three scrambled genes, there may be some partially unscrambled nanochromosomes in the parental macronucleus that generate these small number of micronuclear-specific 27 nt RNAs.

**Table 2:** Venn Diagram analysis of 26–28 nt small RNA alignment to macronuclear/micronuclear sequence pairs - evidence for a macronuclear origin for the mating-specific small RNAs.

Gene	Micro Length	Macro Length	MDSs	Scrambled?	Micro Count	Macro Count	Union Count	Xsect Count	Xsect Pct	Only Micro Count	Only Micro Pct	Only Macro Count	Only Macro Pct
Actin	2115	1503	10	Yes	718	1056	1078	696	64.56%	22	2.04%	360	33.40%
Zinc Finger	2128	1994	4	No	641	681	681	641	94.13%	0	0.00%	40	5.87%
DNAPolAlpha	7165	4645	47	Yes	1326	1771	1826	1271	69.61%	55	3.01%	500	27.38%
L29Cyclo	1811	1596	3	No	500	503	503	500	99.40%	0	0.00%	3	0.60%
TEBPAlpha	2787	2127	17	Yes	744	992	996	740	74.30%	4	0.40%	252	25.30%
TEBPBeta	1753	1250	7	No	612	651	651	612	94.01%	0	0.00%	39	5.99%

Macronuclear sequences and the micronuclear sequences from which they were derived were used as targets for Bowtie mapping. Micro and macronuclear sequence pairs were trimmed so that all macronuclear sequence was contained within the micronuclear clone and regions of the micronuclear clone outside of the macronuclear gene were trimmed. The result of this is that sequence length differences between the micronuclear and macronuclear versions of the gene are due to IESs and repeated pointers in the micronuclear sequence. The gene name, length of the micronuclear and macronuclear sequences used in the filter, the number of MDSs, and whether the order of the MDSs is scrambled in the micronucleus are indicated. The number of distinct 26–28 nt small RNA sequences from each of the mating-derived small RNA libraries that aligned to each sequence in the pair were determined and then added together (“MicroCount” and “MacroCount”). “UnionCount” is the sum of the distinct sequences in each library found in the union of MicroCount and MacroCount. “XsectCount” is the number of distinct 26–28 nt sequences that aligned to both the macronuclear and micronuclear targets for that gene, and “XsectPct” is the percentage of UnionCount found in XsectCount. “OnlyMicroCount” is the number of distinct sequences in the seven libraries that only align to the micronuclear gene sequence and “OnlyMicroPct” is the percentage of sequences in UnionCount that are found in OnlyMicroCount. “OnlyMacroCount” is the number of distinct sequences in the seven libraries that only align to the macronuclear gene sequence and “OnlyMacroPct” is the percentage of sequences in UnionCount that are found in OnlyMacroCount.

### Visualization of small RNA coverage

In order to visualize the coverage of 26–28 nt RNAs on the macronuclear genome, we employed a minimal build of the UCSC Genome Browser [33] on an Ubuntu Linux computer. We made an *Oxytricha trifallax* genome consisting of two chromosomes, chr1 and chr2, which we call “oxytri1”. chr1 corresponds to the concatenated complete nanochromosomes and chr2 corresponds to the concatenated partially-assembled nanochromosomes that were used in our Bowtie macronuclear alignments (Table 1, Figures 3 and 4). Using mapping of the reads in Bam format [34], we are able to visualize alignments of 26–28 nt small RNA sequencing reads to their (possibly multiple) alignment sites on the assembled contigs.

**Figure 6:**
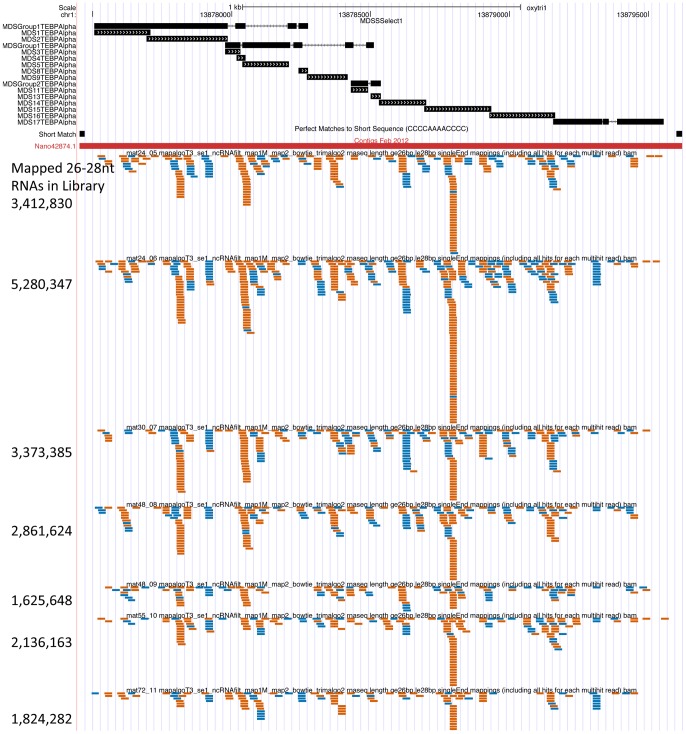
27 nt RNA reads from different mating libraries align to both strands of a nanochromosome. This screen shot from the *Oxytricha trifallax* macronuclear genome build of the UCSC Genome Browser shows the macronuclear nanochromosome corresponding to the alpha telomere binding protein gene indicated as a red bar (Nano42874.1) with the telomere sequences at the beginning and end of the nanochromosome noted as small black boxes in the track above that. The top track shows the locations of groups of MDS sequences or individual MDSs, as aligned using BLAT of the micronuclear sequence against the macronuclear genome. The alignments of 26–28 nt small RNAs from 7 different mating small RNA libraries (mat24_05, mat24_06, mat30_07, mat48_08, mat48_09, mat55_10 and mat72_11 - see Table 1) are shown with alignments to the plus strand of the nanochromosome indicated in blue and alignments to the minus strand of the nanochromosome indicated in orange. Numbers at the left indicate the total number of 26, 27 and 28 nt RNA sequences from each of the seven libraries that mapped to the macronuclear genome.

Figure 6 shows a screen shot from the *Oxytricha trifallax* macronuclear genome browser with the alignment of 26–28 nt sequencing reads from 7 different mating libraries to the alpha telomere binding protein macronuclear gene. All the libraries have 27 nt sequences that originate from both strands of the nanochromosome. There appears to be a non-random distribution of reads, with pileups at specific places that are maintained in all the libraries and which are specific to each strand. In order to give a sense for coverage density relative to total reads in each library, the total number of 26–28 nt reads from each library that mapped to the macronuclear genome is indicated at the left of the figure.

**Figure 7:**
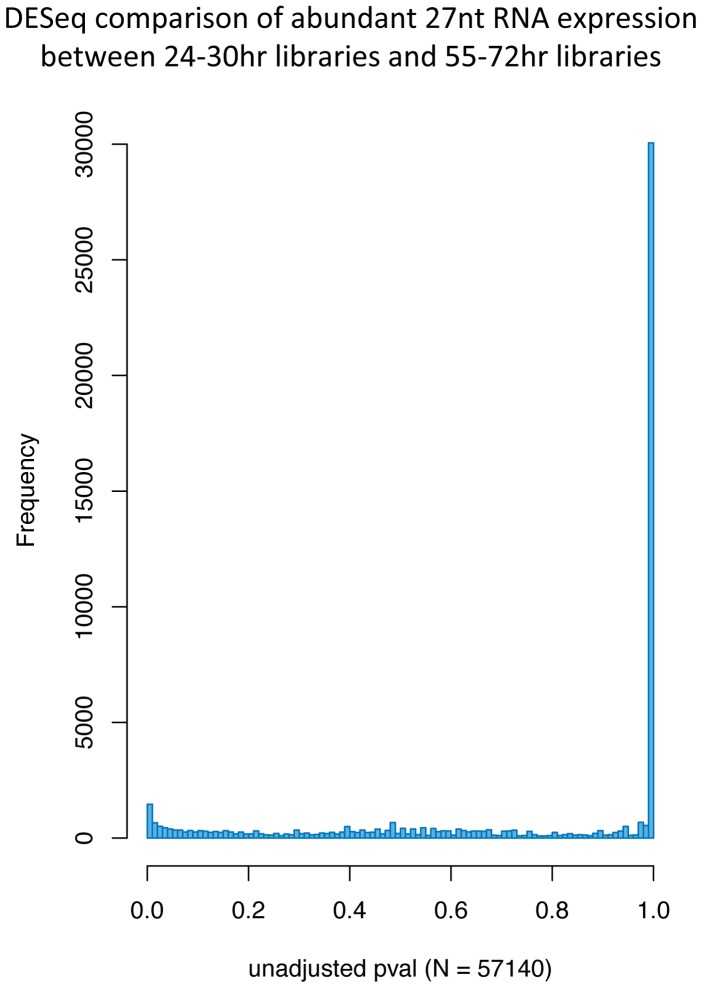
Histogram of unadjusted p-values for changes in relative expression level for 27 mers between early and late mating libraries. DESeq [35] was used to compare relative expression levels of distinct 27 mers whose sequence was found 10 or more times in any of the mating libraries. The relative expression of each of 57,140 abundant 27 mers between early mating time points (mat24_05, mat24_06 and mat30_07) and later mating time points (mat55_10 and mat72_11) was compared. Unadjusted p-values for changes in expression for each of the 27 mers were plotted on the histogram.

To test whether there is evidence to suggest that any of the small RNA sequences that appear in multiple copies in the different libraries decrease or increase in number at different rates relative to the other sequences during macronuclear development, we analyzed the data using DESeq [35]. For all distinct 27 mers whose sequence occurred at least 10 times in *any* of the seven libraries, we counted the number of occurrences of that sequence in each of the libraries. We compared the expression of these 27 mers between the early mating libraries (24–30hrs - mat24_05, mat24_06 and mat30_07) and the later mating libraries (55–72hrs - mat55_10 and mat72_11). DESeq determined an unadjusted p-value for the difference in relative expression of each of the 27 mers between these two sets of libraries. Figure 7 shows a histogram of these p-values. For 52.6% of the 27 mers examined, the unadjusted p-value for these two expression conditions is between 0.99 and 1.0, indicating that the relative expression of these 27 mers does not change during macronuclear development. A small fraction of abundant 27 mers did show significant changes (even after adjusting for multiple hypothesis testing) during macronuclear development, and these outliers will be interesting for further study. Based on our ^32^P labeling of total RNA during a developmental time course (Figure 1), we know that the total amount of the 27 mer pool peaks 24 hours from the start of mating and decreases as macronuclear development proceeds. We hypothesize that for the majority of mating-specific 27 mers, their abundance decreases at a uniform rate as macronuclear development proceeds. This is in contrast to the scanRNAs of *Tetrahymena* which undergo a population filtering step during macronuclear development [4], [14].

**Figure 8:**
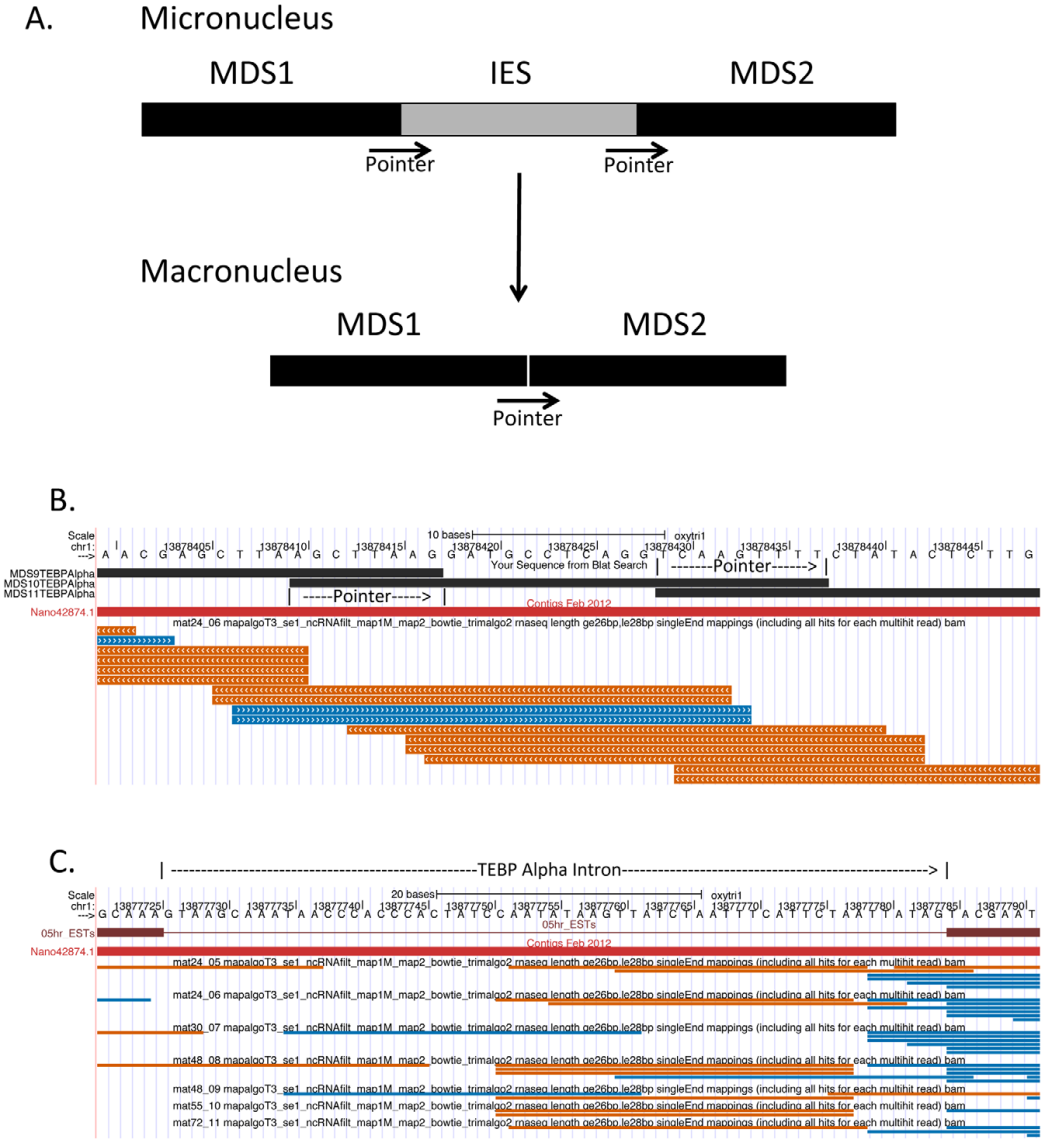
27 nt small RNAs are macronuclear in origin and do not require intron removal for their generation. **A**. Schematic diagram of internally eliminated sequence (IES) removal and macronuclear destined sequence (MDS) joining during macronuclear development. Pointer sequences are direct repeats found in the micronucleus at MDS borders. Only one copy of the pointer is found in the macronucleus. **B**. *Oxytricha trifallax* Macronuclear Genome Browser screen shot of a region of the alpha telomere binding protein gene showing the junctions and overlapping pointer sequences of MDSs 9, 10 and 11. Only the mat24_06 library 26–28 nt small RNA track is shown. Note that the 26–28 nt small RNAs from the mat24_06 library from both strands overlap the MDS junction and pointer sequences. This is consistent with these small RNAs having originated from the mature parental macronucleus. **C**. Screen shot of a region of the alpha telomere binding protein gene containing its intron. 26–28 nt small RNAs from all seven mating libraries are shown. Note that sense strand small RNAs are found within the intron, indicating that they could be processed from introns. Also, small RNAs in the sense strand are found crossing the downstream intron/exon border, indicating that these are made from an RNA that did not undergo intron processing.

From the small RNA sequence alignment to alpha telomere binding protein in Figure 6, it is clear that sequence reads are found that correspond to each of the 17 MDS sequences whose location is indicated at the top of the figure. As seen in Figure 8B, some of these reads cross the MDS/MDS junctions and include sequences on both sides of the pointers as predicted by the analysis of the data in Table 2. An interesting question is whether the 27 nt small RNAs that correspond to the plus strand of the open reading frame are produced from processed mRNA or perhaps the pre-mRNA or some other precursor specific to small RNA production in which the introns are not removed by splicing. Compared to other eukaryotes, introns are relatively rare in *Oxytricha trifallax* [8]. One intron is present in the alpha telomere binding protein pre-mRNA (Figure 8C). For this intron and others not shown here, there is evidence of plus strand 27 nt RNAs derived from the intron, and from the intron/exon junctions. This indicates that a least a subset of the 27 nt RNAs from this nanochromosome are derived from RNAs in which introns are not removed.

### The 27 nt RNAs arise from both strands and are not uniformly distributed

**Figure 9:**
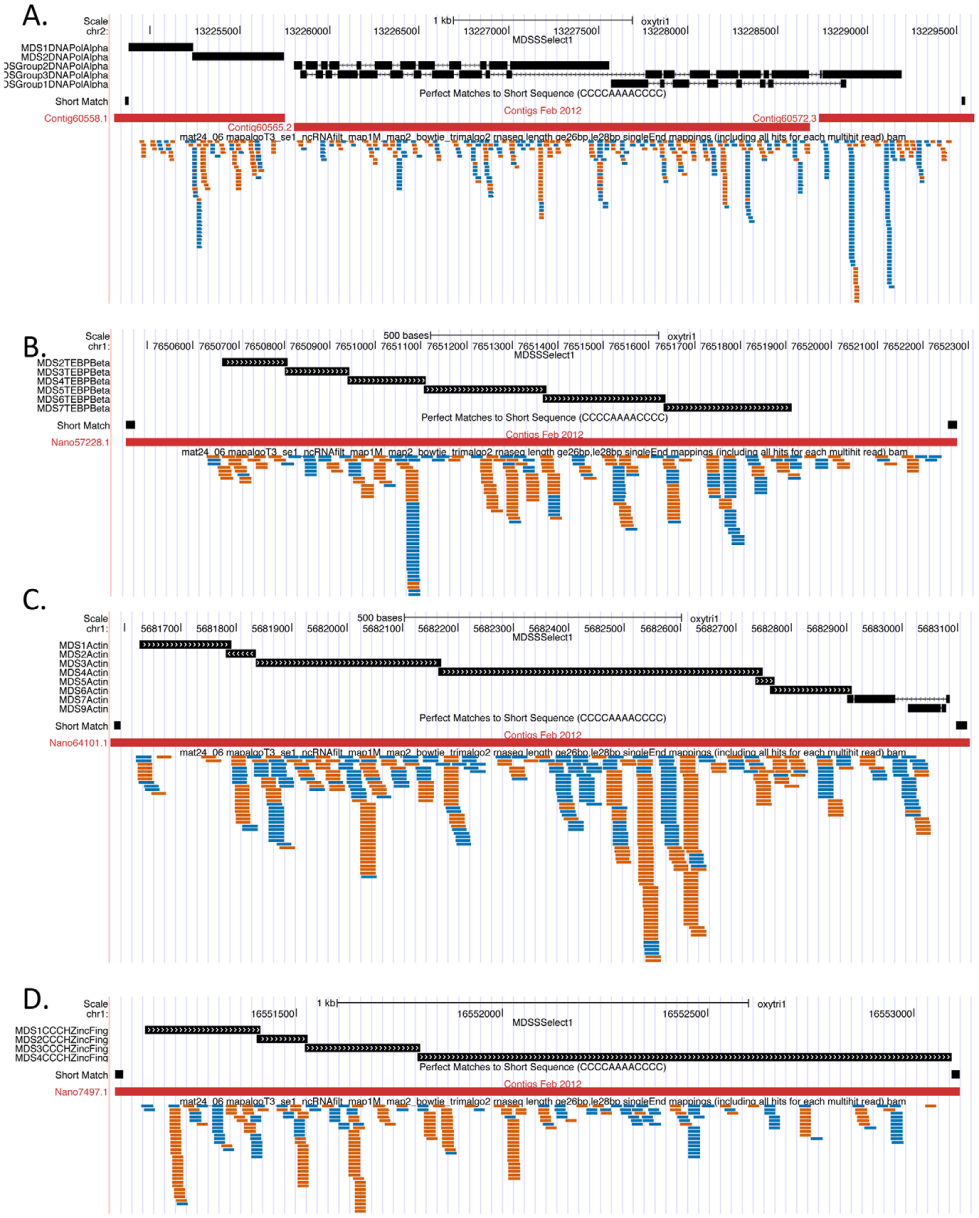
Screen shots of four different macronuclear genes from the *Oxytricha* macronuclear genome browser. In each panel, black boxes on top are a BLAT alignment of individual MDS sequences to show MDS junction location. Short match below that shows telomeric repeat locations. The red bar below that shows the nanochromosome contig extent. Blue and orange bars indicate the alignment location of 26–28 nt long small RNAs from the mat24_06 library. Plus strand alignments are in blue and minus strand alignments are in orange. **A**. DNA Polymerase Alpha. Note that this nanochromosome is incompletely assembled and is spread across three partially assembled contigs on chr2. **B**. Beta Telomere Binding Protein. **C**. Actin-I. **D**. CCCH Zinc Finger protein.

**Figure 10:**
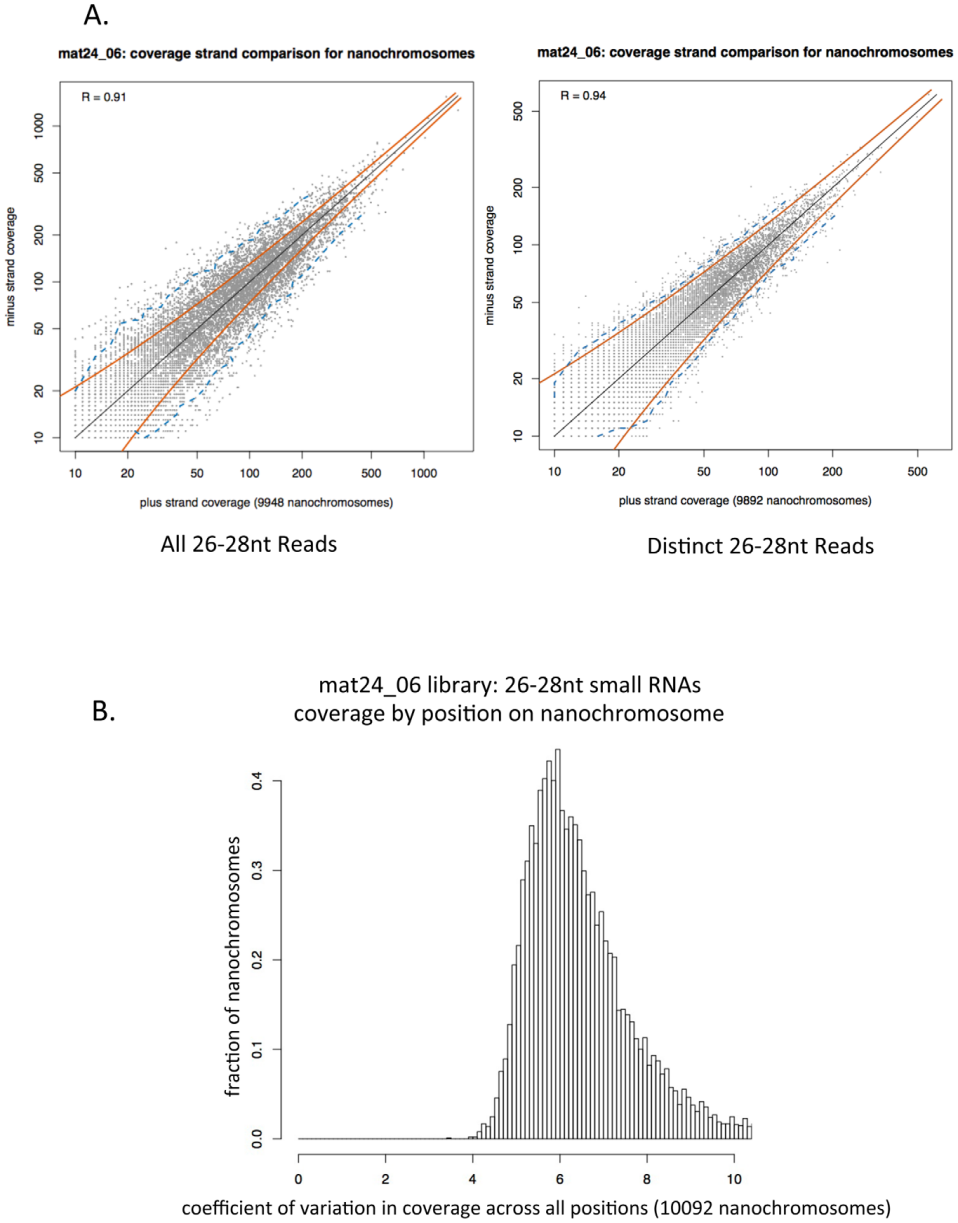
Analysis of the distribution of 27 nt RNAs on macronuclear nanochromosomes. **A**. 27 nt RNA small RNAs are produced in equal numbers from both strands of the nanochromosome. For each full length nanochromosome with at least ten 26–28 nt small RNAs aligning to each strand, the total number of 26–28 nt small RNAs that map to each strand was plotted (left graph) or the number of distinct 26–28 nt small RNAs that map to each strand was plotted (right graph). Pearson R correlation values of 0.91 for all reads and 0.94 for distinct reads were obtained. This indicates a strong correlation of small RNA production from one strand of the nanochromosome with production of small RNAs from the other strand of the nanochromosome. Red curved lines represent 2 standard deviations from the mean; 95% of points would be expected to fall within these regions if there is a one-to-one correlation between the number of 26–28 nt small RNAs aligning to each strand of a nanochromosome (thin black line along the main diagonal). The blue dotted line indicates the actual lines within which 95% of the data points fall. **B**. There is a non-uniform distribution in the positioning of small RNAs on the nanochromosomes. For each position in each complete nanochromosome, the number of 26–28 nt small RNAs that start at that position in the mat24_06 sequencing library were determined. Then we determined the mean and standard deviation of coverage density on the nanochromosome. The coefficient of variation (standard deviation of coverage divided by the mean coverage) is plotted for each nanochromosome in the histogram. The peak of coefficient of variation at ∼6.0 indicates that the standard deviation is 6.0 times greater than the mean. This is highly indicative of a non-uniform distribution of small RNA coverage on the nanochromosomes.

Figure 9 shows genome browser screen shots of 26–28 nt RNA coverage of 4 additional nanochromosomes. These nanochromosomes all have micronuclear gene sequence available, and the locations of the MDSs are indicated at the top of each panel. This figure supports the conclusion that the 27 nt RNA species is produced from both strands of the nanochromosomes and that they do not have a uniform distribution across those strands. In order to determine how well-correlated the production of 27 nt RNAs are from both strands of the macronuclear genome, we plotted the number of 26–28 nt reads derived from each strand for each nanochromosome that had 10 or more 26–28 nt RNAs align to it. In Figure 10A we plotted the total number of reads from the mat24_06 library that align to each strand of each nanochromosome (these read alignment counts include multiple identical reads of the same sequence - left side of the figure) and we plotted the number of distinct reads that align to each strand (only one occurrence of a sequence that occurs multiple times in a library was used - right side of the figure). Pearson correlations (R) of 0.91 and 0.94 are obtained for the two plots, indicating a strong correlation between the numbers of small RNAs matching the two different strands of each nanochromosome. The red lines in each plot represent predicted positions of two standard deviations from the mean; in a model where a read is equally likely to arise from either strand, 95% of points should fall between these lines. The blue dotted lines represent the measured region in which 95% of the points lie. As can be seen, plotting distinct read coverage allows for an almost perfect fit to the theoretical curve, while plotting the total reads for each strand allows for more variation than expected. This difference may be related to the fact that we generally do not see a uniform distribution of read density along the nanochromosomes, and we observe many piles of identical reads that align to either strand. To quantify this effect, we calculated the coefficient of variation (standard deviation divided by mean) of the counts of 26–28 nt reads across the positions of each complete nanochromosome in chr1. The distribution of this statistic over all the nanochromosomes has a peak at about 6.0 or greater (depending on the library). Figure 10B shows a graph of coefficient of variance for 26–28 nt RNAs in the mat24_06 library. The peak at 6.0 (standard deviation of the coverage of 26–28 nt RNAs at any position on a nanochromosome is 6 times greater than the mean coverage at any position on that nanochromosome), is consistent with a highly non-uniform distribution of reads across the nanochromosome. This effect is not due to insufficient coverage, as when we calculate the coefficient of variation measurement for the top 10% of nanochromosomes with the highest 26–28 nt RNA coverage, we still obtain a coefficient of variation for the mat24_06 library of 5.80 (data not shown). From the plots in Figure 10, we conclude that there is a strong correlation between the two strands of the nanochromosomes in terms of the number of 26–28 nt sequence reads derived from each strand and that the coverage of reads on each strand is highly non-uniform.

### The 27 nt RNA class has lower coverage in the telomere-proximal region

**Figure 11:**
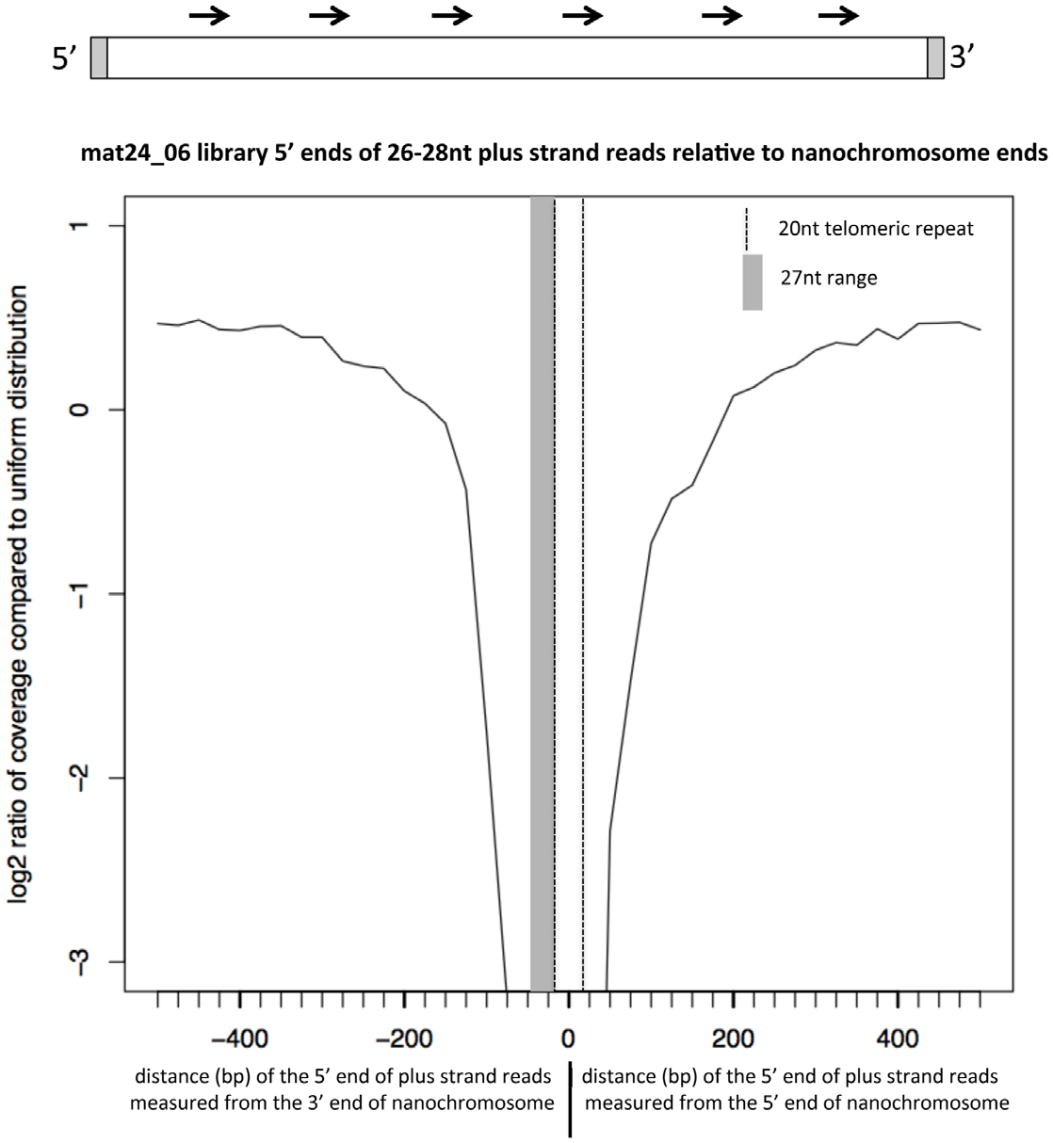
The first 30 nt of the nanochromosomes proximal to the telomeres have >8-fold lower coverage of 26–28 nt small RNAs relative to the rest of the nanochromosome. Graphic at the top shows a nanochromosome (rectangle) with 20 nt telomere sequence in gray shadow. 27 nt small RNAs for the plus strand of the nanochromosome are indicated as arrows above the nanochromosome. The average density of the location of 5′ ends of plus strand-aligning 26–28 nt RNAs was determined over 500 positions (in 20 bins of 25 bp each, shown by tick marks) relative to the 5′ (right side, positive values) and 3′ (left side, negative values) ends of 10,002 complete nanochromosomes. These densities were plotted relative to a uniform distribution of the aligned 26–28 nt RNAs over the same sets of 500 positions on the same nanochromosomes. A dotted line at 20 nt from either end is included on both sides of the zero point to indicate the average telomere length of 20 nt on the nanochromosome sequences to which these data were plotted. The shaded gray area, 27 nt wide, is included on the plot from the nanochromosome 3′ end because the plus strand-aligning reads would have 27 nt of sequence between their plotted 5′ end and the 3′ end of the nanochromosome. When minus strand-aligning reads were analyzed by this same method, a mirror image plot was obtained that is otherwise identical to the graph for plus end reads (not shown).

In addition to the non-uniformity in read coverage across entire nanochromosomes, we have observed that small telomere-proximal regions of the nanochromosomes are strongly depleted for the 27 nt small RNAs, aligned to either strand, relative to the rest of the nanochromosome. To quantify this, we determined the average 26–28 nt coverage density of each of 10,002 complete nanochromosomes from chr1 that were at least 500 bp in length. For each of these nanochromosomes, we measured the density of 5′ ends of 26–28 nt small RNAs at each of the proximal 500 nt positions from the 5′ or 3′ end of the nanochromosome, for both the plus strand or minus strand-aligning reads. These distributions were plotted relative to a plot of uniform distribution based on the average coverage over the 500 positions at the appropriate end of the nanochromosome (Figure 11). The telomere repeat in the nanochromosome sequences in the chr1 collection is on average 20 nt, and is indicated by a dashed vertical line in the figure. As small RNA sequences made up of the telomeric repeat would have been filtered out as part of the initial non-coding RNA filter step, we expect to have no coverage in this region. It should be noted that only 237 reads out of 10,570,000 total sequences applied to the non-coding RNA filter in the mat24_06 library matched the telomeric repeat, so the non-coding RNA filter step cannot explain the paucity of coverage of small RNAs at the telomere and subtelomeric regions. Since the counts of the 5′ ends of the RNAs were plotted, a shaded region 27 nt long was added to the plot for the plus strand reads measured from the 3′ end of the nanochromosome to account for the 27 nt read length. When these data are plotted, it is clear that there is a zone of 30 nt proximal to the telomere that has over 8-fold lower density of 27 nt RNA coverage relative to a uniform distribution. We did identical plots of the minus strand-aligning 26–28 nt RNAs for mat24_06, and found that the graph obtained is a mirror-image of the one shown in Figure 11 (data not shown), again indicating that the 30 bp proximal to the telomere on the nanochromosome have over 8-fold lower small RNA coverage than the rest of the nanochromosome. The WGS2.1.1 full-length nanochromosome sequence collection has no information regarding which strand contains an open reading frame, but given our results here and the fact that both strands of nanochromosomes are transcribed to generate the 27 mers (Figure 10A), there seems to be no correlation between the coding strand and the lack of 27 nt RNA coverage near the telomeres.

## Discussion

In ciliates, there is a clear role for the parental macronucleus to provide epigenetic information to control the complex DNA elimination and rearrangements involved in the development of the new macronucleus. This epigenetic information is carried in the form of RNA [14]. We explored the production of small RNAs in *Oxytricha trifallax* during mating and subsequent macronuclear development. We have identified an RNA species of 27 nt in length whose production is induced upon mating. Next generation sequencing and analysis of these RNAs demonstrates that they are macronuclear in origin. We have decided to name these “27macRNAs”, because of their length and their origin in the parental macronucleus.

The potential biogenesis of the 27macRNAs is interesting. They are transcribed from both strands of the nanochromosomes, indicating a requirement for an RNA polymerase activity that recognizes a common feature of both strands of the nanochromosomes. A good candidate for such a common feature is the telomeres. We had previously identified an RNA polymerizing activity from vegetative macronuclei of *Oxytricha nova* that could use telomeric extensions and their internal double-stranded sequences as templates [36]. At the time, we proposed that this activity was responsible for creating the RNA primers for DNA replication at the telomeres (a DNA primase), but this activity also fits the requirements of an enzyme that can transcribe both strands of the nanochromosomes from the telomeres.

We propose that the 27macRNAs are processed from long double-stranded RNAs by a dicer-like activity, similar to the micronuclear-derived scanRNAs in Tetrahymena [16], [18]. Our observation of the presence of a 5′ monophosphate and 3′ OH group on the 27macRNAs is consistent with dicer products. It has been reported in *Oxytricha trifallax* that there is bidirectional transcription of long RNAs from the nanochromosomes 6 hours after mating strains are mixed together. These have been hypothesized to guide macronuclear rearrangements [23]. The timing of production of these bi-directional long RNAs occurs prior to the appearance of 27macRNAs. We hypothesize that these may serve as precursors for 27macRNA production by a dicer family enzyme. In *Drosophila*, some classes of piRNAs are found matching both genomic strands and associate with distinct PIWI proteins for each strand [37]. The ping-pong model for production of these small RNAs from both strands holds that piRNAs processed from one strand direct cleavage of complementary piRNAs from the other strand between nucleotides 10 and 11 of the cleaving piRNA. This cleavage results in half of the piRNAs having an A at the tenth nt position, which is complementary to a U at the 5′ end of the partner piRNA that directed its cleavage [38]. We see no evidence for an A bias in the 27 mers at position 10 in all distinct 27macRNAs (Figure 5D) nor even in abundant 27macRNAs (Figure 5E), nor any evidence of 10 base overlap of 27macRNAs on opposite strands, so it is unlikely that the ping-pong model can explain bidirectional production of piRNAs.

Our observation that the subtelomeric regions of the nanochromosomes have much lower coverage of 27macRNAs from either strand relative to the rest of the nanochromosome has several possible explanations. In one theory, RNA polymerase would generate a long RNA from each strand of a nanochromosome, with subsequent pairing of the complementary strands to form a long double-stranded RNA molecule. An inability of the RNA polymerase activity that makes these longer 27mac precursors to begin transcription at the very beginning of the nanochromosomes, or an inability of this enzyme to copy RNA to the very 3′ end of the nanochromosomes, would lead to single-stranded regions at the ends of the double-stranded RNA that result from pairing of the two long complementary RNA strands. These single-stranded regions would not be substrates for processing by the double-stranded endonuclease activity of dicer. Whatever the reason for low representation of 27macRNAs in subtelomeric regions, the lack of coverage of 27macRNAs in this region rules out the possibility that these RNAs play a direct role in guiding the precise *de novo* addition of telomeres to the nanochromosome ends in the developing macronucleus. However, their absence from the region may be important, allowing for the enzymes that cleave and add telomeres to function at the sites of precise telomere addition.

Epigenetic information from the parental macronucleus, sent through an RNA intermediate, has been hypothesized to control three different aspects of macronuclear development in stichotrichous ciliates. These are (1) the joining of MDSs and importantly the unscrambling of the micronuclear genome [23], (2) the control of macronuclear nanochromosome copy number [24], [25], and (3) the control of chromatin formation in the developing macronucleus which may play a role in guiding DNA elimination [32]. The 27macRNAs may be involved in several of these processes. Long RNAs made bi-directionally from the nanochromosomes in the parental macronucleus have been observed and have been demonstrated experimentally to function in guiding unscrambling of the macronuclear genome [23]. These long RNAs may possibly serve as precursors to the 27macRNAs. While 27macRNAs have been observed that cross MDS/MDS junctions, it is unclear whether the population that span MDS junctions are present in sufficient quantities, and contain sufficient information, to serve as guides for MDS joining and unscrambling. We see a wide range in depth of coverage of the different nanochromosomes by the 27macRNAs (Figure 10A), and it is possible that the copy number of these nanochromosomes could be reflected in the number of 27macRNAs produced from each nanochromosome. If that is the case, then the 27macRNAs could be the RNA species that provide information to the developing macronucleus on the copy number of individual nanochromosomes in the parental macronucleus [24], [25].

Another potential function of 27macRNAs is to direct chromatin modifications in the developing macronucleus. A thorough analysis of chromatin modifications present in the developing macronucleus in the stichotrichous ciliate *Stylonichia* has been performed [32]. Postberg et al. found that during macronuclear development, sequences to be retained in the mature macronucleus are associated with permissive histone chromatin modifications, while sequences to be eliminated are associated with repressive histone modifications. They also identified a Piwi-family protein, Piwi/mdp1, whose accumulation in different nuclear types changes during the course of macronuclear development, and which is associated with chromatin in the macronuclear anlage [32]. Piwi proteins bind small RNAs and can affect heterochromatin [4], [39], so a satisfying hypothesis is that 27macRNAs function to target the macronuclear-destined regions in the anlagen for protection from DNA elimination by altering their chromatin state. Given that nucleosome core protects ∼146 bp of DNA, this potential role for 27macRNAs would exclude a role for them in guiding MDS joining as the IESs to be eliminated are smaller than the amount of DNA associated with the nucleosome.

Alternative models for 27macRNA function can be proposed in which this class of RNAs do an important job during macronuclear development but do not provide epigenetic information. They may function to change the chromatin state of the parental macronucleus in which they are made, helping to down-regulate gene expression from the parental macronucleus. They may act as siRNAs, targeting mRNAs from the parental macronucleus for destruction. They may function to promote DNA elimination during destruction of the parental macronucleus. Further studies to identify the protein binding partners of the 27macRNAs and to determine the subcellular localization of these RNAs during macronuclear development will be required to shed light on a functional role for the 27macRNAs.

Note: Wenwen Fang and colleagues (Princeton, USA) have shared with us that they have also observed production of 27nt-long mating-specific RNAs of macronuclear origin in *Oxytricha trifallax* (L.F. Landweber, personal communication - Wenwen Fang, Xing Wang, John Bracht, Mariusz Nowacki, and Laura F. Landweber, manuscript submitted).

## Materials and Methods

### Vegetative growth of *Oxytricha trifallax*

*Oxytricha trifallax* strains JRB310 and JRB510 [40], [41] were kindly provided by Robert O. Hammersmith (Ball State University). Vegetative growth of cells was carried out in Pyrex dishes in inorganic salts media [42] using the algae *Chlorogonium elongatum* (UTEX Collection Strain B203) as a food source.

### Mating of Oxytricha trifallax

Mating competent strains were grown separately until they had nearly exhausted their food. Cells were filtered through cotton to remove algal debris and were then concentrated on 10 µM Nitex filters. Cells were washed off of the filters into inorganic salts media and the individual cultures were counted. Cells of the different mating strains were mixed together in equal numbers, concentrated again on Nitex filters and washed on the filters into Pringsheim salts buffer. Timing of mating begins at this mixing step. Cells were put into Pyrex dishes at a concentration of 1200–2000 cells/ml in Pringsheim solution with 1 ml of stationary *Klebsiella pneumoniae* culture per 300 ml of mating cells as food. Cells began to show strong levels of aggregation by 5 hours and mating pairs were first visible after 8 hours. In order to refresh the JRB310 and JRB510 stocks to provide for a more robust mating, exconjugants with anlage were individually isolated from a JRB310 x JRB510 mating and these clonal isolates were grown as cultures. After six weeks of vegetative growth, we tested the mating compatibility of each of 12 clonal cultures by pairwise mixing. Two of the new strains, ALXC2 and ALXC9 showed strong mating ability with each other, and their progeny had high survival rates. ALXC2 cultures have a tendency to self mate if starved, and surprisingly these self-progeny are also viable. No ALXC9 selfing is evident. When equal numbers of ALXC2 and ALXC9 are mixed together, a highly efficient mating occurs, with ∼70% of cells possessing anlage 48 hours after mixing.

### Total RNA isolation

*Oxytricha trifallax* cultures were washed through cotton and then concentrated on 10 µM Nitex filters. Cells were put into microcentrifuge tubes and gently pelleted at 1000 x g for 2 minutes. Supernatants were removed except for the 50 µl above the cell pellet. 200 µl of mirVana Lysis/Binding Buffer from the mirVana miRNA Isolation Kit (Ambion) was added to each tube. Total RNA was purified according to the kit's protocols for total RNA purification (not the miRNA purification protocol). Typical total RNA yields from this protocol starting with 300 ml of *Oxytricha trifallax* culture were 100 µg.

### ^32^P labeling of total RNA

3 µg of total RNA were treated with 30 units of calf intestine alkaline phosphatase (New England Biolabs) in a 100 µl reaction mixture using the manufacturer's protocol. The reaction mixture was acid phenol:CHCl3 extracted, Na-Acetate added to 0.3 M and ethanol precipitated with 4 volumes of ethanol and 1 µl glycoblue (Ambion) as carrier. Precipitate pellets were washed with 70% ethanol, dried and then resuspended in 10 µl of deionized H_2_O. ^32^P labeling was done using T4 polynucleotide kinase (NEB) and gamma-^32^P-ATP in 10 µl reaction mixtures. An equal volume of formamide dyes were added to the reaction mixtures and then heated to 95°C. Labeled RNA samples were separated on 40 cm long 15% acrylamide (19∶1) urea gels in TBE buffer. ^32^P labeled Decade marker (Ambion) was used for a 10 nt ladder size reference. Gel images were recorded using a Typhoon PhosphorImager (GE Health Care) and ImageQuant software.

### Beta elimination assay

The beta elimination assay was used to test for modifications at the 3′ end of RNAs. ^32^P labeled total RNA was prepared and reaction mixtures were raised to 95°C for 2 minutes after the kinase reaction in order to inactivate the T4 polynucleotide kinase enzyme. In addition, 250 pmoles of a 21 nt synthesized RNA with the *C. elegans* lin-4 microRNA sequence and 250 pmoles of a 23 nt synthesized RNA with the *C. elegans* mir-90 sequence were ^32^P labeled at their 5′ ends in 10 µl reaction mixtures to serve as positive controls for beta elimination, as these synthetic RNAs have a 2′ and 3′ OH group on their 3′ terminal ribose. After labeling and heat inactivation, 1.5 µg of unlabeled total *Oxytricha* RNA was added to each tube so that each control RNA was treated in the beta elimination assay in the same complex RNA background as the labeled total RNA. Beta elimination reaction conditions were essentially those described by Horwich et al. [43]. 5 µl of heat inactivated ^32^P kinase reaction mixture were added to 100 µl of 25 mM Na-meta-periodate in 60 mM Borax/60 mM Boric acid buffer pH 8.6. Tubes were incubated at room temperature for 30 minutes. Then, 13 µl of 1 M NaOH was added to each tube in order to raise the pH from 8.6 to 9.5. Reaction mixtures were then incubated for 90 minutes at 45°C to allow the beta elimination reaction to occur. RNA was recovered after acid phenol:CHCl3 extraction and ethanol precipitation, and dried pellets were raised in 10 µl of formamide loading dye. Products were separated on 15% polyacrylamide urea sequencing gels and visualized by autoradiography.

### Small RNA cDNA Library Preparation

Small RNAs had linkers ligated to them and bar-coded cDNAs were prepared using the TruSeq Small RNA Sample Prep Kit (Illumina) following the manufacturer's instructions. Library preparation began with 1 µg of total RNA from mating cells in which small RNAs are abundant, or 2 µg of total RNA from vegetative cells in which small RNAs (especially the 27 nt species) are rare. Individual libraries were analyzed on a BioAnalyzer (Agilent) for the presence of linkered cDNA at the appropriate size (135–165 bp) and 11 bar-coded libraries were pooled into one sample by mixing 2.0 ng of the 135–165 bp peak from each sample as determined by the BioAnalyzer. The 135–165 bp peak of the pooled cDNAs was purified from the mixed sample using the Pippin Prep DNA Size Selection System (Sage Science), and confirmed by BioAnalyzer.

### Illumina Sequencing

Sequencing of the pooled libraries was performed in one lane of the Illumina HiSeq2000 Sequencer at the UCSC Genome Technology Center. 100 bp paired-end reads of the libraries were obtained. After indexing and trimming of linker sequences, those reads of at least 16 nt in length that had 100% identity in the two directions (77% of the total) were further analyzed.

### Non-coding RNA filter

We extracted *Oxytricha trifallax* non-coding RNA sequences from the list of non-coding RNAs published by Jung et al. [28]. This included rRNAs, tRNAs, snRNAs, telomerase RNA and other non-coding RNA species. We also included a 40 base telomeric repeat sequence in this filter (GGGGTTTT)_5_ to filter out any RNAs derived from telomeres. This non-coding RNA filter was used as a first pass filter of the libraries prior to alignment to the macronuclear genome.

### Preparation of a macronuclear reference genome

In order to be able to understand where the 27 nt small RNAs originate, an assembly of macronuclear sequences was needed. While the macronuclear genome sequence of *Oxytricha trifallax* is currently incomplete, an extensive assembly of whole genome shotgun sequencing contigs, WGS2.1.1, was released in the supplemental material of Jung et al. [28]. From that data we extracted the sequences of 10,137 full-length telomere-to-telomere nanochromosome sequences. We concatenated these nanochromosome sequences together into one file with 50 Ns inserted in between each full-length nanochromosome. This file is referred to as chr1 and contains only full-length nanochromosomes. An additional 46,417 contigs of incomplete nanochromosome sequence were extracted from WGS2.1.1 and these were also concatenated with 50 Ns inserted in between each contig. This collection of partial nanochromosome sequences is referred to as chr2. We noted that there is a small amount of gene duplication on chr1 (∼5%) and that there is a good deal of sequence duplication within the incomplete contigs on chr2 and between chr1 and chr2. The 70 kb *Oxytricha trifallax* mitochondrial genome sequence [44] was used to analyze small RNAs (referred to as chrM). chr1 and chr2 were also used as reference genome sequences in a build of a minimal UCSC Genome Browser [33], [45] on a local Linux computer, which allowed for visualization of Illumina sequencing reads through Bam mapping [34] and BLAT functionality [46].

### Analysis Pipeline

Bowtie [47] was used to first align the sequencing reads (trimmed and selected for equality of the two ends as noted above) from each library to the non-coding RNA filter described above. The reads that were not caught by this filter were aligned to the concatenated full-length nanochromosomes sequences (chr1) and to chrM. The remaining unmapped reads were aligned to the concatenated partial nanochromosomes (chr2). More stringent criteria were used for the mapping to the chromosomes (Bowtie parameters: -n 1 –e 60) than were used for the filtering of ncRNA reads (Bowtie parameters: -n 3 –e 150). For reads that mapped to multiple locations, a single, randomly selected, mapping was retained for the subsequent statistical analysis. For visualization of libraries on the genome browser, all mappings of multi-hit reads were retained. For the comparison of micronuclear and macronuclear origins of the RNA, the reads that were not caught by the non-coding RNA filter were mapped to the “micro/macro” reference described below, with all mappings retained.

### Alignment of 27 nt small RNAs to micronuclear/macronuclear sequence pairs

In order to determine whether the 27 nt small RNAs were macronuclear or micronuclear in origin, we compared 26–28 nt sequence reads that passed through the non-coding RNA filter for the ability to align to a micronuclear version of a gene as compared to its macronuclear counterpart. To do this, we used the micronuclear and macronuclear versions as Bowtie filters for the sequence reads, and identified and counted the number of sequence reads that aligned to each set in the pair. This allowed us to create a Venn diagram (Table 2) of small RNAs that aligned to both the micronuclear and macronuclear versions, only the micronuclear version or only the macronuclear version. Gene pairs tested in this assay were trimmed so that all macronuclear sequence was contained within the micronuclear clone, and the regions of the micronuclear clone outside of the macronuclear sequences were removed. Essentially, the only differences between the micronuclear and macronuclear sequences were the presence/absence of IESs and the scrambled nature of the MDS order for some of the genes tested. Sequences used for this experiment were as follows: DNA Polymerase Alpha - micronuclear GenBank accession DQ525914.1 nucleotides 1837-9001 and macronuclear GenBank accession U59426.1 nucleotides 20-4665; Actin-I - micronuclear GenBank accession U19288.1 nucleotides 1-2115 and macronuclear GenBank accession HQ432909.1 nucleotides 1-1503; Zinc Finger gene - micronuclear GenBank accession FJ346576.1 nucleotides 1-2128 and macronuclear GenBank accession FJ346577.1 nucleotides 90-2084; L29Cyclo - micronuclear GenBank accession DQ081723.1 nucleotides 1-1811 and macronuclear GenBank accession DQ081724.1 nucleotides 21-1616; TEBPAlpha - micronuclear GenBank accession EU047939.1 nucleotides 1-2787 and macronuclear GenBank accession EU047938.2 nucleotides 21-2147, TEBPBeta - micronuclear GenBank accession EU047941.2 nucleotides 1-1753 and macronuclear GenBank accession EU047940.2 nucleotides 1–1250.

### Availability of sequence data

Raw sequence files for all 11 Illumina sequencing libraries, and files listing the sequences of 26–28 nt RNAs that survived the non-coding RNA filter from each of the 7 mating libraries, have been deposited in GEO - Accession Number GSE37390.

## References

[1] Prescott DM (2000) Genome gymnastics: unique modes of DNA evolution and processing in ciliates. Nat Rev Genet 1: 191–198. 11252748 10.1038/35042057

[2] Jahn CL, Klobutcher LA (2002) Genome remodeling in ciliated protozoa. Annu Rev Microbiol 56: 489–520. 12142486 10.1146/annurev.micro.56.012302.160916

[3] Nowacki M, Landweber LF (2009) Epigenetic inheritance in ciliates. Curr Opin Microbiol 12: 638–643. 19879799 10.1016/j.mib.2009.09.012 PMC2868311

[4] Chalker DL, Yao MC (2011) DNA elimination in ciliates: transposon domestication and genome surveillance. Annu Rev Genet 45: 227–246. 21910632 10.1146/annurev-genet-110410-132432

[5] Lauth MR, Spear BB, Heumann J, Prescott DM (1976) DNA of ciliated protozoa: DNA sequence diminution during macronuclear development of Oxytricha. Cell 7: 67–74. 820431 10.1016/0092-8674(76)90256-7

[6] Swanton MT, Heumann JM, Prescott DM (1980) Gene-sized DNA molecules of the macronuclei in three species of hypotrichs: size distributions and absence of nicks. DNA of ciliated protozoa. VIII. Chromosoma 77: 217–227. 6771110 10.1007/BF00329546

[7] Prescott DM, Prescott JD, Prescott RM (2002) Coding properties of macronuclear DNA molecules in Sterkiella nova (Oxytricha nova). Protist 153: 71–77. 12022278 10.1078/1434-4610-00084

[8] Cavalcanti AR, Stover NA, Orecchia L, Doak TG, Landweber LF (2004) Coding properties of Oxytricha trifallax (Sterkiella histriomuscorum) macronuclear chromosomes: analysis of a pilot genome project. Chromosoma 113: 69–76. 15258807 10.1007/s00412-004-0295-3

[9] Prescott DM, DuBois ML (1996) Internal eliminated segments (IESs) of Oxytrichidae. J Eukaryot Microbiol 43: 432–441. 8976601 10.1111/j.1550-7408.1996.tb04502.x

[10] Greslin AF, Prescott DM, Oka Y, Loukin SH, Chappell JC (1989) Reordering of nine exons is necessary to form a functional actin gene in Oxytricha nova. Proc Natl Acad Sci U S A 86: 6264–6268. 2503830 10.1073/pnas.86.16.6264 PMC297818

[11] Mitcham JL, Lynn AJ, Prescott DM (1992) Analysis of a scrambled gene: the gene encoding alpha-telomere-binding protein in Oxytricha nova. Genes Dev 6: 788–800. 1577273 10.1101/gad.6.5.788

[12] Hoffman DC, Prescott DM (1996) The germline gene encoding DNA polymerase alpha in the hypotrichous ciliate Oxytricha nova is extremely scrambled. Nucleic Acids Res 24: 3337–3340. 8811087 10.1093/nar/24.17.3337 PMC146089

[13] Kataoka K, Mochizuki K (2011) Programmed DNA elimination in Tetrahymena: a small RNA-mediated genome surveillance mechanism. Adv Exp Med Biol 722: 156–173. 21915788 10.1007/978-1-4614-0332-6_10 PMC3766321

[14] Nowacki M, Shetty K, Landweber LF (2011) RNA-Mediated Epigenetic Programming of Genome Rearrangements. Annu Rev Genomics Hum Genet 12: 367–389. 21801022 10.1146/annurev-genom-082410-101420 PMC3518427

[15] Chalker DL, Yao MC (2001) Nongenic, bidirectional transcription precedes and may promote developmental DNA deletion in Tetrahymena thermophila. Genes Dev 15: 1287–1298. 11358871 10.1101/gad.884601 PMC313804

[16] Mochizuki K, Gorovsky MA (2005) A Dicer-like protein in Tetrahymena has distinct functions in genome rearrangement, chromosome segregation, and meiotic prophase. Genes Dev 19: 77–89. 15598983 10.1101/gad.1265105 PMC540227

[17] Mochizuki K, Gorovsky MA (2004) Conjugation-specific small RNAs in Tetrahymena have predicted properties of scan (scn) RNAs involved in genome rearrangement. Genes Dev 18: 2068–2073. 15314029 10.1101/gad.1219904 PMC515285

[18] Malone CD, Anderson AM, Motl JA, Rexer CH, Chalker DL (2005) Germ line transcripts are processed by a Dicer-like protein that is essential for developmentally programmed genome rearrangements of Tetrahymena thermophila. Mol Cell Biol 25: 9151–9164. 16199890 10.1128/MCB.25.20.9151-9164.2005 PMC1265777

[19] Mochizuki K, Fine NA, Fujisawa T, Gorovsky MA (2002) Analysis of a piwi-related gene implicates small RNAs in genome rearrangement in tetrahymena. Cell 110: 689–699. 12297043 10.1016/s0092-8674(02)00909-1

[20] Yao MC, Chao JL (2005) RNA-guided DNA deletion in Tetrahymena: an RNAi-based mechanism for programmed genome rearrangements. Annu Rev Genet 39: 537–559. 16285871 10.1146/annurev.genet.39.073003.095906

[21] Liu Y, Taverna SD, Muratore TL, Shabanowitz J, Hunt DF, et al (2007) RNAi-dependent H3K27 methylation is required for heterochromatin formation and DNA elimination in Tetrahymena. Genes Dev 21: 1530–1545. 17575054 10.1101/gad.1544207 PMC1891430

[22] Taverna SD, Coyne RS, Allis CD (2002) Methylation of histone h3 at lysine 9 targets programmed DNA elimination in tetrahymena. Cell 110: 701–711. 12297044 10.1016/s0092-8674(02)00941-8

[23] Nowacki M, Vijayan V, Zhou Y, Schotanus K, Doak TG, et al (2008) RNA-mediated epigenetic programming of a genome-rearrangement pathway. Nature 451: 153–158. 18046331 10.1038/nature06452 PMC2647009

[24] Heyse G, Jonsson F, Chang WJ, Lipps HJ (2010) RNA-dependent control of gene amplification. Proc Natl Acad Sci U S A 107: 22134–22139. 20974970 10.1073/pnas.1009284107 PMC3009782

[25] Nowacki M, Haye JE, Fang W, Vijayan V, Landweber LF (2010) RNA-mediated epigenetic regulation of DNA copy number. Proc Natl Acad Sci U S A 107: 22140–22144. 21078984 10.1073/pnas.1012236107 PMC3009799

[26] Kurth HM, Mochizuki K (2009) 2′-O-methylation stabilizes Piwi-associated small RNAs and ensures DNA elimination in Tetrahymena. RNA 15: 675–685. 19240163 10.1261/rna.1455509 PMC2661841

[27] Yang Z, Vilkaitis G, Yu B, Klimasauskas S (2007) Chen× (2007) Approaches for studying microRNA and small interfering RNA methylation in vitro and in vivo. Methods Enzymol 427: 139–154. 17720483 10.1016/S0076-6879(07)27008-9 PMC3574582

[28] Jung S, Swart EC, Minx PJ, Magrini V, Mardis ER, et al (2011) Exploiting Oxytricha trifallax nanochromosomes to screen for non-coding RNA genes. Nucleic Acids Res 39: 7529–7547. 21715380 10.1093/nar/gkr501 PMC3177221

[29] Pak J, Fire A (2007) Distinct populations of primary and secondary effectors during RNAi in C. elegans. Science 315: 241–244. 17124291 10.1126/science.1132839

[30] Sijen T, Steiner FA, Thijssen KL, Plasterk RH (2007) Secondary siRNAs result from unprimed RNA synthesis and form a distinct class. Science 315: 244–247. 17158288 10.1126/science.1136699

[31] Adl SM, Berger JD (2000) Timing of life cycle morphogenesis in synchronous samples of Sterkiella histriomuscorum. II. The sexual pathway. J Eukaryot Microbiol 47: 443–449. 11001141 10.1111/j.1550-7408.2000.tb00073.x

[32] Postberg J, Heyse K, Cremer M, Cremer T, Lipps HJ (2008) Spatial and temporal plasticity of chromatin during programmed DNA-reorganization in Stylonychia macronuclear development. Epigenetics Chromatin 1: 3. 19014664 10.1186/1756-8935-1-3 PMC2603335

[33] Kent WJ, Sugnet CW, Furey TS, Roskin KM, Pringle TH, et al (2002) The human genome browser at UCSC. Genome Res 12: 996–1006. 12045153 10.1101/gr.229102 PMC186604

[34] Li H, Handsaker B, Wysoker A, Fennell T, Ruan J, et al (2009) The Sequence Alignment/Map format and SAMtools. Bioinformatics 25: 2078–2079. 19505943 10.1093/bioinformatics/btp352 PMC2723002

[35] Anders S, Huber W (2010) Differential expression analysis for sequence count data. Genome Biol 11: R106. 20979621 10.1186/gb-2010-11-10-r106 PMC3218662

[36] Zahler AM, Prescott DM (1989) DNA primase and the replication of the telomeres in Oxytricha nova. Nucleic Acids Res 17: 6299–6317. 2475856 10.1093/nar/17.15.6299 PMC318279

[37] Siomi MC, Sato K, Pezic D, Aravin AA (2011) PIWI-interacting small RNAs: the vanguard of genome defence. Nat Rev Mol Cell Biol 12: 246–258. 21427766 10.1038/nrm3089

[38] Brennecke J, Aravin AA, Stark A, Dus M, Kellis M, et al (2007) Discrete small RNA-generating loci as master regulators of transposon activity in Drosophila. Cell 128: 1089–1103. 17346786 10.1016/j.cell.2007.01.043

[39] Lin H, Yin H (2008) A novel epigenetic mechanism in Drosophila somatic cells mediated by Piwi and piRNAs. Cold Spring Harb Symp Quant Biol 73: 273–281. 19270080 10.1101/sqb.2008.73.056 PMC2810500

[40] Williams K, Doak TG, Herrick G (1993) Developmental precise excision of Oxytricha trifallax telomere-bearing elements and formation of circles closed by a copy of the flanking target duplication. EMBO J 12: 4593–4601. 8223469 10.1002/j.1460-2075.1993.tb06148.x PMC413894

[41] Zoller SD, Hammersmith RL, Swart EC, Higgins BP, Doak TG, et al.. (2012) Characterization and Taxonomic Validity of the Ciliate Oxytricha trifallax (Class Spirotrichea) Based on Multiple Gene Sequences: Limitations in Identifying Genera Solely by Morphology. Protist. 10.1016/j.protis.2011.12.006 PMC3433844 22325790

[42] Chang WJ, Stover NA, Addis VM, Landweber LF (2004) A micronuclear locus containing three protein-coding genes remains linked during macronuclear development in the spirotrichous ciliate Holosticha. Protist 155: 245–255. 15305799 10.1078/143446104774199628

[43] Horwich MD, Li C, Matranga C, Vagin V, Farley G, et al (2007) The Drosophila RNA methyltransferase, DmHen1, modifies germline piRNAs and single-stranded siRNAs in RISC. Curr Biol 17: 1265–1272. 17604629 10.1016/j.cub.2007.06.030

[44] Swart EC, Nowacki M, Shum J, Stiles H, Higgins BP, et al (2012) The Oxytricha trifallax mitochondrial genome. Genome Biol Evol 4: 136–154. 22179582 10.1093/gbe/evr136 PMC3318907

[45] Fujita PA, Rhead B, Zweig AS, Hinrichs AS, Karolchik D, et al (2011) The UCSC Genome Browser database: update 2011. Nucleic Acids Res 39: D876–882. 20959295 10.1093/nar/gkq963 PMC3242726

[46] Kent WJ (2002) BLAT–the BLAST-like alignment tool. Genome Res 12: 656–664. 11932250 10.1101/gr.229202 PMC187518

[47] Langmead B, Trapnell C, Pop M, Salzberg SL (2009) Ultrafast and memory-efficient alignment of short DNA sequences to the human genome. Genome Biol 10: R25. 19261174 10.1186/gb-2009-10-3-r25 PMC2690996

